# Machine learning-based analysis of [^18^F]DCFPyL PET radiomics for risk stratification in primary prostate cancer

**DOI:** 10.1007/s00259-020-04971-z

**Published:** 2020-07-31

**Authors:** Matthijs C. F. Cysouw, Bernard H. E. Jansen, Tim van de Brug, Daniela E. Oprea-Lager, Elisabeth Pfaehler, Bart M. de Vries, Reindert J. A. van Moorselaar, Otto S. Hoekstra, André N. Vis, Ronald Boellaard

**Affiliations:** 1 Amsterdam UMC, Vrije Universiteit Amsterdam, Department of Radiology and Nuclear Medicine, Cancer Center Amsterdam, De Boelelaan, 1117 Amsterdam, the Netherlands; 2 Amsterdam UMC, Vrije Universiteit Amsterdam, Department of Urology, Cancer Center Amsterdam, De Boelelaan, 1117 Amsterdam, the Netherlands; 3 Amsterdam UMC, Vrije Universiteit Amsterdam, Department of Epidemiology and Biostatistics, De Boelelaan, 1117 Amsterdam, the Netherlands; 4 Department of Nuclear Medicine and Molecular Imaging, Medical Imaging Center, University of Groningen, Groningen, the Netherlands

**Keywords:** Machine learning, Prostate cancer, PSMA PET-CT, Radiomics

## Abstract

**Purpose:**

Quantitative prostate-specific membrane antigen (PSMA) PET analysis may provide for non-invasive and objective risk stratification of primary prostate cancer (PCa) patients. We determined the ability of machine learning-based analysis of quantitative [^18^F]DCFPyL PET metrics to predict metastatic disease or high-risk pathological tumor features.

**Methods:**

In a prospective cohort study, 76 patients with intermediate- to high-risk PCa scheduled for robot-assisted radical prostatectomy with extended pelvic lymph node dissection underwent pre-operative [^18^F]DCFPyL PET-CT. Primary tumors were delineated using 50–70% peak isocontour thresholds on images with and without partial-volume correction (PVC). Four hundred and eighty standardized radiomic features were extracted per tumor. Random forest models were trained to predict lymph node involvement (LNI), presence of any metastasis, Gleason score ≥ 8, and presence of extracapsular extension (ECE). For comparison, models were also trained using standard PET features (SUVs, volume, total PSMA uptake). Model performance was validated using 50 times repeated 5-fold cross-validation yielding the mean receiver-operator characteristic curve AUC.

**Results:**

The radiomics-based machine learning models predicted LNI (AUC 0.86 ± 0.15, *p* < 0.01), nodal or distant metastasis (AUC 0.86 ± 0.14, *p* < 0.01), Gleason score (0.81 ± 0.16, *p* < 0.01), and ECE (0.76 ± 0.12, *p* < 0.01). The highest AUCs reached using standard PET metrics were lower than those of radiomics-based models. For LNI and metastasis prediction, PVC and a higher delineation threshold improved model stability. Machine learning pre-processing methods had a minor impact on model performance.

**Conclusion:**

Machine learning-based analysis of quantitative [^18^F]DCFPyL PET metrics can predict LNI and high-risk pathological tumor features in primary PCa patients. These findings indicate that PSMA expression detected on PET is related to both primary tumor histopathology and metastatic tendency. Multicenter external validation is needed to determine the benefits of using radiomics versus standard PET metrics in clinical practice.

**Electronic supplementary material:**

The online version of this article (10.1007/s00259-020-04971-z) contains supplementary material, which is available to authorized users.

## Introduction

In primary prostate cancer (PCa), risk stratification is crucial to determine prognosis and treatment strategies. Extended pelvic lymph node dissection (ePLND) is the current standard for identification of lymph node metastases [1–3]. This procedure, however, is invasive and associated with complications such as lymphocele, venous thrombosis, and extended hospital stays [4, 5]. Hence, patients at risk for lymph node involvement (LNI) are selected using clinical nomograms, but these lack adequate performance [3]. Also, histopathology data (e.g., Gleason score, GS) used as input for these nomograms are based on error-prone prostate biopsies [6]. Taken together, a novel biomarker able to pre-operatively stratify high- and low-risk patients is highly needed.

Prostate-specific membrane antigen (PSMA) is a type-II transmembrane protein known to be highly overexpressed on PCa cells [7]. Kaittanis et al. demonstrated that PSMA is a stimulator of oncogenic signaling, clarifying the role of PSMA in PCa progression [8]. Moreover, primary tumor PSMA expression on immunohistochemistry was shown to have prognostic value [9–11]. Therefore, quantitative measures of PSMA expression are promising biomarkers for risk stratification of primary PCa patients.

PSMA expression may be quantified non-invasively using PSMA ligand positron emission tomography computed tomography (PET-CT). A novel approach for quantification is to use radiomics analysis, which entails high-throughput image data mining aiming to capture a tumor’s phenotype and perhaps its metastatic tendency [12–14]. Machine learning can be employed to translate the high-dimensional radiomics data into clinically actionable predictions [15]. In contrast with tumor biopsies, radiomics may characterize the local tumor phenotype based on the entire lesion instead of through tumor subsamples.

We investigated whether machine learning-based analysis of quantitative [^18^F]DCFPyL PET-CT data predicts metastatic disease and high-risk tumor features in patients with intermediate- and high-risk primary PCa scheduled to undergo robot-assisted radical prostatectomy and ePLND. Predictions using a full radiomics feature set were compared to those based on standard PET metrics only, and the influence of tumor delineation and partial-volume correction (PVC) was evaluated.

## Materials and methods

### Patients

Seventy-six consecutive patients underwent pre-operative [^18^F]DCFPyL PET-CT for staging purposes in a prospective cohort study (NL6754). We analyzed patients included between November 2017 and August 2019. Inclusion criteria were (1) biopsy-proven prostate adenocarcinoma and (2) clinical indication for robot-assisted radical prostatectomy with ePLND based on either an ≥ 8% risk score of LNI based on the Memorial Sloan Kettering Cancer (MSKCC) nomogram or any high-risk feature (≥ T3, Gleason > 7, PSA > 20 ng/mL). Patients with distant metastases on PET for whom surgery was omitted were only included in case of histopathological confirmation. Only patients who underwent [^18^F]DCFPyL PET-CT at the Amsterdam UMC were included. Surgical tissue specimens (prostate and lymph nodes) were reviewed according to international guidelines by uropathologists [3]. The Amsterdam UMC medical ethical committee provided formal approval (2017.543) and patients provided written informed consent.

### Outcomes

All references outcomes were pathology-proven, and dichotomized for machine learning-based classification: post-operative GS (< 8 versus ≥ 8), presence of extracapsular tumor extension (ECE; ≤ pT2b versus ≥ pT3a), pathology-proven LNI (N0 versus N1), and presence of any metastasis (pN0 and cM0 versus pN1 and/or pM1). Of note, the “any metastasis” outcome is an expansion of patients with LNI to include patients with distant metastases.

### PET-CT imaging

Patients were scanned on a time-of-flight PET-CT system (Ingenuity, Philips Healthcare) with European Association of Nuclear Medicine Research Ltd. (EARL) accreditation [16]. A CT scan was acquired at 120 kV and 30–110 mAs. Next, whole-body PET was performed at 122.5 ± 11.1 min post-injection of 310.1 ± 16.2 MBq [^18^F]DCFPyL, from mid-thighs to skull base, at 4 min per bed position. Images were reconstructed using iterative ordered subset expectation maximization reconstruction (3 iterations, 33 subsets) with 4-mm voxel dimensions, with corrections for decay, scatter, random coincidences, and attenuation correction. Lucy-Richardson iterative deconvolution (10 iterations) was applied for PVC [17]. The full width at half max for PVC was calibrated at 7.0 mm using a NEMA NU2 Quality Phantom, such that signal recovery was in line with EARL2 guidelines [18]. Original and PVC images were analyzed separately.

### Tumor delineation

An experienced nuclear medicine physician (DO) reviewed all [^18^F]DCFPyL PET-CT scans for intra-prostatic tumor localization. A mask was manually drawn around PET avid intra-prostatic tumor volumes to constrain region-growing and prevent inclusion of bladder activity. All masks were reviewed by a second observer. If needed, consensus was reached through joint revision. Next, tumors were delineated using a region-growing algorithm with a background-adapted peak threshold [17]. The thresholds were varied incrementally from 50 to 70% (5% intervals). Delineation was performed on original and PVC scans separately to mimic clinical practice.

### Radiomics extraction

Radiomic features were extracted from the delineated tumors following descriptions of the Image Biomarker Standardization Initiative using the RaCaT software [19, 20]. Voxel values were scaled to the net injected tracer dosage per kilogram bodyweight (standardized uptake value, SUV). Image voxels and volumes of interest were resampled to 2 × 2 × 2 mm isotropic voxels using tri-linear interpolation as recommended [21, 22]. Per tumor we extracted 480 radiomic features (Supplemental Table 1) on intensity (*n* = 50), morphology (*n* = 22), and texture (*n* = 408). Intensity features encompassed peak intensity, intensity-based statistics, intensity-volume histograms, and intensity histograms. 2D and 3D textural features based on gray-level co-occurrence matrices (GLCM), gray-level run length matrices (GLRLM), gray-level size zone matrices (GLSZM), gray-level distance zone matrices (GLDZM), neighborhood gray-tone difference matrices (NGTDM), and neighboring gray-level dependence matrices (NGLDM) were extracted. Before textural feature calculation, images were discretized using a fixed bin width of 0.25 SUV starting at SUV_min_ [21]. During radiomics extraction, we also derived standard PET features SUV_mean_, SUV_peak_, SUV_max_, PSMA-positive tumor volume, and PSMA-total lesion uptake (the product of SUV_mean_ and volume) and used these data separately as input for the machine learning pipeline.

### Machine learning

**Fig. 1:**
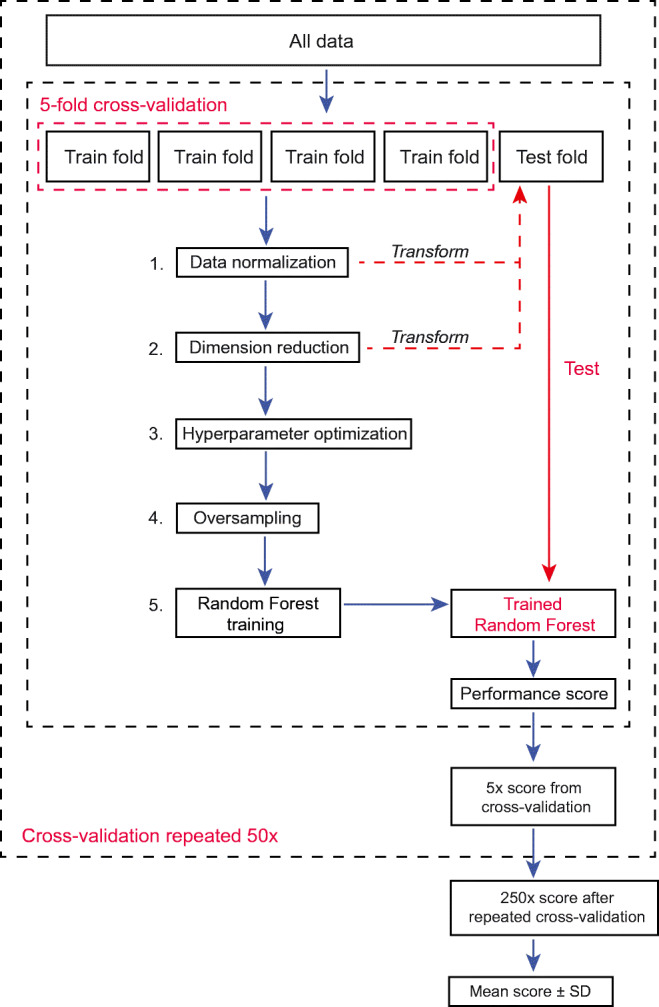
Schematic overview of the implemented machine learning pipeline. Data pre-processing and model tuning are performed on the training dataset in repeated cross-validation to prevent leakage of information between training and testing data

Machine learning algorithms may handle high-dimensional data and/or data with complex non-linear relations with clinical outcomes. We constructed a machine learning framework in Python 3.6 using *Scikit-learn* library 0.21 (pipeline in Fig. 1) [15, 23]. We used a *Random Forest* classifier (1000 decision trees) which is a commonly used non-parametric ensemble algorithm [24]. To assess model generalizability (i.e., its prediction performance on unseen data), we used a stratified 5-fold cross-validation approach. In each cross-validation fold, the random forest was trained on 80% of samples and validated on an unseen subset of 20% of samples. This was repeated until each fold had served as the test set. Finally, this 5-fold cross-validation was repeated 50 times to further limit chance findings. Features were scaled using a *z*-score normalization. Model hyperparameters (tree depth, splitting criterion) were optimized within each training set in nested cross-validation using a randomized search algorithm. All pre-processing and optimization steps were performed within each training fold to prevent leakage of test data into the trained model (Fig. 1).

#### Dimensionality reduction

To mitigate model overfitting and potentially improve generalizability, we applied three different strategies for dimension reduction that reduced the number of features used as input for the random forests: (i) a principal component analysis (PCA) retaining 95% of the observed variance, (ii) a recursive feature elimination approach using a random forest in nested cross-validation, and (iii) a univariate selection method based on ANOVA testing that retained the top 10 percentile features. Models were also trained without any dimensionality reduction. When using standard PET metrics as model input, no dimension reduction was applied because of the small number of metrics.

#### Oversampling

In case of strong class imbalance, a trained machine learning model may have high accuracy in classifying the majority class, but perform poorly in classifying the minority class. Therefore, oversampling was applied in each training set by generation of “synthetic” samples with interpolated feature values (SMOTE) [25]. Models were also trained without oversampling.

#### Feature importance

To explore feature importance, coefficients representing the relative importance of each feature within a trained random forest model can be derived (the sum of coefficients being equal to 1.0). Per outcome, we visualized the top 10% coefficients (*n* = 48) from a random forest trained on the entire dataset using the feature selection method that yielded the highest predictions per outcome (excluding PCA as this does not yield interpretable features).

### Statistical analysis

To evaluate model performance, we generated the receiver-operator characteristic curve and calculated the area under the curve (AUC). The Brier score was used to assess model calibration and refinement (0.0 being optimal) [26]. For each score, we calculated the mean with standard deviation over the repeated cross-validation folds.

Random permutations were performed to test whether the models performed significantly better than random guessing. To this end, labels were randomly shuffled before performing 10 times repeated 5-fold cross-validation, resulting in a “random guessing” cross-validated mean AUC. This was repeated 100 times, yielding a *p* value defined as the fraction of repeated cross-validation iterations in which the permutation mean AUC was equal or higher than the actual mean AUC [27].

Comparing the cross-validated AUCs of machine learning models is a known difficulty due to the complex relations between the trained models and the inherent dependency of train-test iterations [28]. Still, to be able to compare the mean AUCs of radiomics versus standard PET metrics, we used a framework proposed by Van De Wiel et al. [29]: In each fold, the AUCs of two models were compared statistically using DeLong test [30], and the median of the *p* values over the different folds was reported as the final *p* value. A disadvantage of this method is that each *p* value is based on the test set of a single fold only (i.e., 20% of data), resulting in a conservative statistical test with low power to detect true differences.

Intraclass correlation coefficients (ICC, 2-way mixed model, absolute agreement) were calculated for each radiomic feature between original versus PVC images (per delineation threshold), and between delineation thresholds (with and without PVC). ICCs were categorized as poor (ICC < 0.5), moderate (0.5 < ICC < 0.75), good (0.75 < ICC < 0.9), or excellent (ICC > 0.9) [31].

## Results

### Patients

Seventy-one out of 76 patients ultimately underwent surgery (Table 1). Six patients had uptake suspicious for distant metastases on PET (*n* = 2 nodal, *n* = 1 bone, *n* = 3 both), all of which were biopsied. In 4 of these patients, biopsies confirmed malignancy and surgery was omitted; in 2 patients (*n* = 1 bone, *n* = 1 nodal lesion), biopsy did not confirm malignancy and surgery was performed as planned. Additionally, 1 patient had biopsy-proven LNI within the ePLND template, but surgery was omitted due to additional PSMA-positive nodal metastases outside the ePLND template. The pathology outcomes are listed in Table 2.

**Table 1:** Patient characteristics

Number of patients	*n* = 76
Age (mean ± SD)	66 ± 6 years
PSA at PET (median, (range))	11 (4–70) ng/ml
ISUP Gleason grade (biopsy)	*n* (%)
Group 1	4 (5.3%)
Group 2	21 (27.6%)
Group 3	19 (25.0%)
Group 4	21 (27.6%)
Group 5	11 (14.5%)
Positive biopsies % (mean ± SD)	54.7% ± 27.3%
Clinical T-stage	*n* (%)
T1c	26 (34.2%)
T2a	24 (31.6%)
T2b	12 (15.8%)
T2c	11 (14.5%)
T3a	3 (3.9%)

**Table 2:** Pathology outcomes. Seventy-one patients underwent robot-assisted radical prostatectomy with ePLND; 1 patient had biopsy-proven LNI but did not undergo surgery; 4 patients did not undergo surgery due to proven distant metastases

	*n* (%)
ISUP Gleason grade	
Group 1 Group 2 Group 3 Group 4 Group 5	1 (1.4%) 27 (38.0%) 24 (33.8%) 5 (7.0%) 14 (19.7%)
Pathological T-stage	
T2a-c T3a-b T4	35 (49.3%) 35 (49.3%) 1 (1.4%)
LNI	
No Yes	62 (86.1%) 10 (13.9%)
Distant metastases	
No Yes	72 (94.7%) 4 (5.3%)
Resection margin status	
R0 R1	43 (60.6%) 28 (39.4%)

### Predictions

**Fig. 2:**
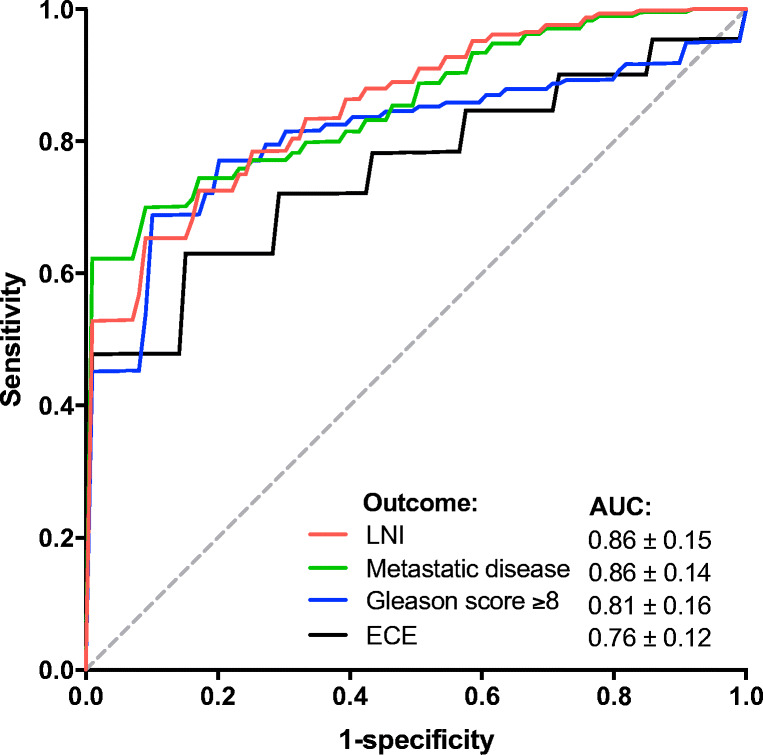
Mean cross-validated ROC curves of radiomics-based machine learning models. Random forest with univariate feature selection and minority class oversampling for LNI, metastasis, and GS prediction. Random forest recursive feature elimination without oversampling for ECE prediction

**Fig. 3:**
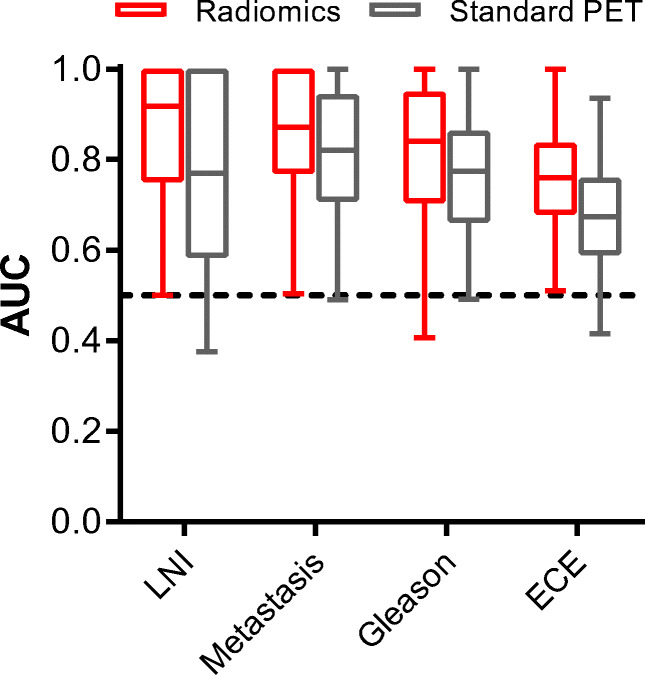
Cross-validation AUCs of the optimal radiomics-based and standard PET metrics-based machine learning models for each outcome of interest

The highest cross-validation mean AUCs of LNI, metastasis, GS, and ECE prediction were 0.86 ± 0.15, 0.86 ± 0.14, 0.81 ± 0.16, and 0.76 ± 0.12, respectively (all *p* < 0.01; Fig. 2). The models using standard PET metrics as input reached lower AUCs with generally larger variability (Fig. 3). These highest mean AUCs were 0.77 ± 0.21 for LNI (*p* = 0.03), 0.81 ± 0.16 for any metastasis (*p* < 0.01), 0.76 ± 0.14 for GS (*p* < 0.01), and 0.67 ± 0.14 (*p* = 0.03) for ECE. Yet, our conservative statistical test was not able to demonstrate significant differences (*p* = 0.25–0.29). The average Brier scores of radiomics-based models were lower (better) than those of standard PET metrics-based models for LNI (0.09 ± 0.05 versus 0.14 ± 0.06), any metastasis (0.10 ± 0.04 versus 0.11 ± 0.04), GS (0.15 ± 0.06 versus 0.17 ± 0.05), and ECE prediction (0.21 ± 0.05 versus 0.24 ± 0.06). Results for all radiomics analyses are presented in Supplemental Table 2.

### Feature importance

**Fig. 4:**
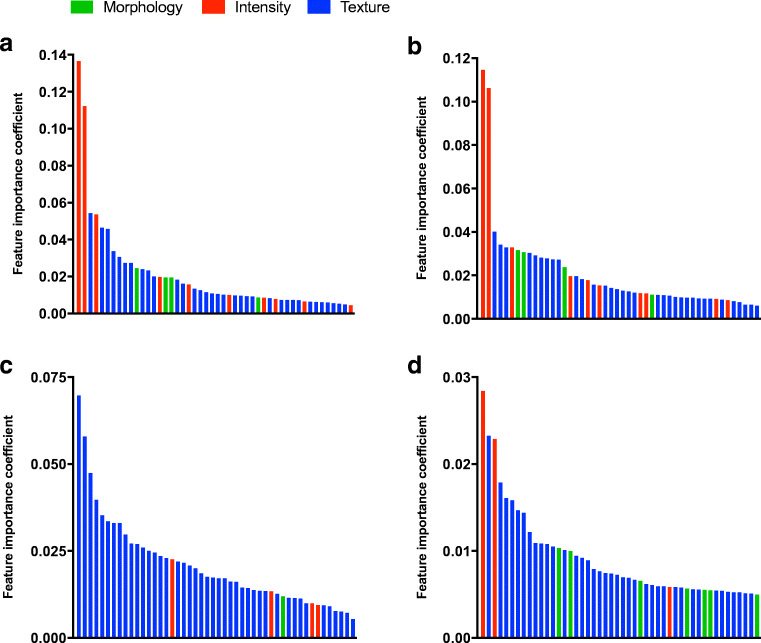
Feature importance coefficients from random forests trained using radiomics to predict **a** LNI, **b** any metastasis, **c** Gleason score ≥ 8, and **d** ECE. Each bar represents the relative feature importance coefficient from a single radiomic feature. Shown are the top 10 percentile feature coefficients

For both LNI and any metastasis prediction, intensity-based features *difference volume at intensity fraction* (importance coefficient 0.14 and 0.11, respectively) and *volume at intensity fraction 10* (importance coefficient 0.11 and 0.11, respectively) were most important, followed by multiple textural features and in a lesser extent several morphological features (Fig. 4). For GS prediction, textural features were evidently most important, specifically *zone size non uniformity* (importance coefficient 0.07), *zone distance non uniformity* (importance coefficient 0.06), and *gray level variance* (importance coefficient 0.05), with minor contributions from intensity and morphological features. For ECE prediction, again the *difference volume at intensity fraction* (importance coefficient 0.03) and *volume at intensity fraction 10* (importance coefficient 0.02) features were among the most important features, along with *gray level non uniformity* (GLSZM; importance coefficient 0.02). The intensity-based features *difference volume at intensity fraction* and *volume at intensity fraction 10* did not correlate with SUVs, volume, and total lesion PSMA uptake from the best models using standard PET metrics (*R*^2^ = 0.00–0.18). The mentioned textural features important for Gleason score and ECE prediction correlated variably with total lesion PSMA uptake (*R*^2^ = 0.28–0.84), and poorly with SUVs and volume (*R*^2^ = 0.10–0.50). See Supplemental Table 3 for individual feature importance coefficient values.

### Impact of PVC and delineation threshold

**Fig. 5:**
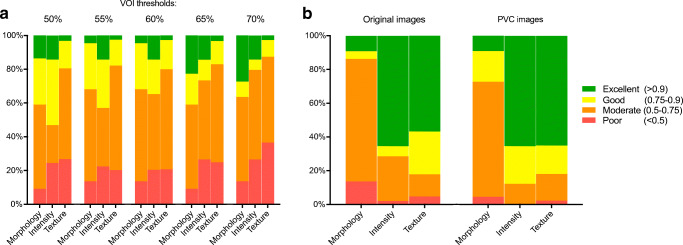
Agreement of radiomic features **a** between original versus PVC images at each delineation threshold, and **b** between the applied delineation thresholds for original and PVC images. Shown are the relative distributions of the radiomics ICC values per ICC category (poor, moderate, good, or excellent)

**Fig. 6:**
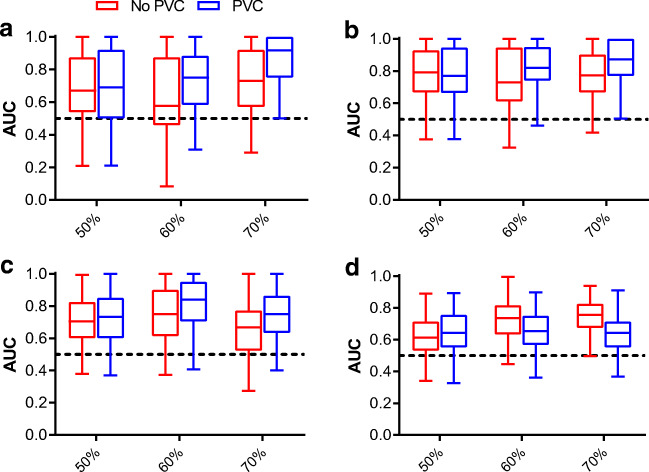
Cross-validation AUCs for each outcome as a function of delineation threshold and use of PVC. **a** LNI, **b** any metastasis, **c** Gleason score ≥ 8, and **d** ECE prediction. For illustrative purposes, results are shown for 50%, 60%, and 70% peak delineation thresholds. Machine learning models using univariate feature selection and oversampling. Boxplots are outlier-trimmed (± 2.5 percentile)

Most radiomic features had a moderate agreement between original and PVC data (Fig. 5a). Delineation thresholds mainly affected morphological features, while intensity and textural features were less affected (Fig. 5b). In terms of their effect on prediction accuracy, PVC and a higher delineation threshold improved model stability for LNI and any metastasis prediction, reducing the width of the cross-validation AUC distributions (Fig. 6a–b). For example, at 50% peak threshold without PVC the lower limit of the cross-validation AUCs was well below 0.5, while at 70% peak with PVC this was not the case (Fig. 6a–b). For GS prediction, PVC benefitted model performance and an intermediate delineation threshold (e.g., 60%) was optimal (Fig. 6c). ECE prediction benefitted from a higher delineation threshold but not from PVC (Fig. 6d). The delineated tumor volumes at each delineation threshold with and without PVC are shown in the Supplemental Figure.

### Impact of data pre-processing

Dimension reduction had a limited effect on mean AUCs, with median differences of − 0.02 (range − 0.11 to 0.07), − 0.02 (range − 0.07 to 0.04), − 0.02 (range − 0.11 to 0.04), and 0.00 (range − 0.11 to 0.04) for LNI, any metastasis, GS, and ECE prediction, respectively. Between the evaluated dimension reduction methods, there was no apparent benefit of using one approach over the other. Oversampling only had a minor impact on AUCs for LNI and metastasis prediction, with a median difference in AUCs of + 0.02 (range − 0.06 to 0.07) and + 0.02 (range − 0.01 to 0.06), respectively. Overall, GS and ECE prediction did not benefit from oversampling, with a median difference in AUCs of 0.0 (ranging − 0.02 to 0.05) and 0.0 (no range), respectively.

## Discussion

**Fig. 7:**
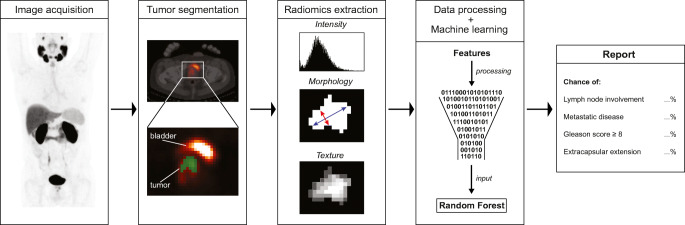
Illustration of a potential workflow for using [^18^F]DCFPyL radiomics and machine learning in a clinical setting

The present study demonstrates that quantitative [^18^F]DCFPyL PET-CT metrics predict disease risk in primary PCa patients, indicating that PSMA expression detected on PET is related to both local tumor histopathology and metastatic tendency. Therefore, these data could be leveraged in clinical practice to identify low-risk patients for whom ePLND will be unnecessary (Fig. 7). Using a higher tumor delineation threshold and PVC is recommended for future studies. Standard PET metrics yielded non-significantly lower AUCs than radiomics-based models for all outcomes, a finding that will warrant confirmation in external validation studies.

Kaittanis et al. observed that PSMA expression on [^68^Ga]PSMA PET/MR correlated with phosphorylation of Akt, a kinase involved in oncogenic signaling that drives PCa progression, but less so with GS and PSA [8]. This might explain why intensity-based features were most important in prediction of LNI (Fig. 4). Moreover, a recent study observed that PSMA expression on [^68^Ga]PSMA PET correlated with genomic index lesions [32]. While PSMA expression correlated with GS on immunohistochemistry, the association between PSMA uptake on PET (expressed in SUV_max_) and GS is not fully evident [33–35]. This may indicate that information on the spatial distribution of PSMA expression is needed. Indeed, textural features appeared to be most important within the random forest models for GS prediction (Fig. 4). As texture on PET may be partly related to total tumor PSMA uptake, some caution regarding interpretation of these data is warranted. Taken together, PSMA PET radiomics may capture tumor aggressiveness by carrying genomic as well as histopathological information. A full head-to-head comparison of radiomics with genomic, molecular (e.g., PSMA- and androgen receptor expression [36]), and histopathological features will be necessary to establish the biological basis of PSMA PET radiomics.

Zamboglou et al. similarly investigated use of [^68^Ga]PSMA PET radiomics (without machine learning) for prediction of GS ≥ 8 and LNI, observing similar validation AUCs for GS (AUC 0.84) and LNI prediction (AUC 0.85) [37]. However, no cross-validation was applied to prevent chance findings potentially induced by a limited sample size. Also, the authors selected a single radiomic feature for LNI prediction based on its correlation with GS, which might explain why the AUCs of LNI and GS prediction were similar. Ferraro et al. recently evaluated whether standard PET metrics from [^68^Ga]PSMA could predict LNI, and observed AUCs of 0.70–0.76, similar to the AUCs we observed for standard PET metrics [38]. Combined with our findings, these data indicate that the value of quantitative PET data in primary prostate cancer may be valid for both [^18^F]- and [^68^Ga]-labeled PSMA ligands.

Validation of radiomics for predictive modeling warrants that methodological PET factors are taken into account [39]. Theoretically, PVC could improve accuracy of intensity feature measurements in small and heterogeneous lesions, and improve textural features calculation by reducing spill-over between voxels. Conversely, as PVC tends to increase image noise levels, it may also hamper precision of the calculated features, which will especially pertain to those based on texture. As PVC increases tumor-to-background contrast, it may improve tumor delineation, which may be of particular benefit for low-grade prostate cancer lesions that tend to be less avid on PSMA PET. To date, use of PVC is not often considered in PET radiomics studies. Hatt et al. demonstrated that for [^18^F]FDG PET in esophageal cancer, PVC and delineation method did not affect the predictive value of textural features, despite an effect on absolute reads [40]. In our study on [^18^F]DCFPyL in primary prostate cancer, we observed that PVC had a substantial impact on most radiomics features and that delineation threshold mainly affected morphological features (Fig. 5). In terms of outcome predictions, a higher (70%) delineation threshold was beneficial for LNI, metastasis, and ECE prediction and PVC benefitted model performance for LNI, metastasis, and GS prediction (Fig. 6). Taken together, in order to facilitate radiomics analysis, it may be an option to extract radiomics features using a 70%peak threshold on PVC images for all outcomes, as overall this seemed to be the most beneficial approach.

Some studies have observed that in radiomics analyses, calculation of textural features might be biased in small tumors or provide little added value above lesion volume itself [41, 42], suggesting small lesions might need to be excluded from such studies. Still, the redundancy of those features will depend on a complex relationship between lesion size distributions, level of correlation between the individual features, and the relative importance of those features within the prediction models. Perhaps, a better approach to determine the clinical added value of small tumor PET radiomics might be to determine its predictive value and benchmark this against that of basic PET features including volume. Also, a potential benefit of PVC needs to be considered. Despite analyzing predominantly small lesions, we did find significant predictive value in the radiomics data, with higher mean AUCs than those derived using standard PET metrics. Still, future multicenter external validation is needed to demonstrate true benefits of PSMA radiomics over standard PET metrics in these small prostate cancer lesions, especially since using different PET systems with potentially different imaging protocols might negatively affect radiomics-based predictions more than those based on standard PET features.

Our study has several limitations. First, the dataset was relatively small. Still, the significant high cross-validated prediction scores indicate that even for such a training dataset size the machine learning models were able to identify high-risk patients in independent data. Secondly, an external dataset for validation was not yet available. Third, comparing cross-validation scores of radiomics-based versus standard PET metrics-based models proved difficult due to a lack of available statistical tests designed to compare cross-validation scores with adequate power. In the required external model validation, performance of radiomics-based models can be directly compared to performance of basic PET features-based models trained on the current dataset, allowing for standard statistical testing.

## Conclusions

Machine learning-based analysis of quantitative [^18^F]DCFPyL PET data can predict LNI and high-risk pathological tumor features in patients with primary PCa. These data demonstrate that the spatial distribution and levels of PSMA expression quantified on [^18^F]DCFPyL PET may be related to both tumor histopathological grade and metastatic tendency. Our results suggest that the performance of radiomics-based analysis is at least equivalent to that of standard PET metrics, while radiomics features can be generated at no additional cost (i.e., from the same analysis pipeline as standard features). External and multicenter validation of the models trained on the current dataset is needed to determine the net benefits of using radiomics versus standard PET metrics in clinical practice.

## Electronic supplementary material

ESM 1 (PDF 45 kb).

ESM 2 (PDF 570 kb).

ESM 3 (PDF 135 kb).

ESM 4 (PDF 136 kb).

## Data Availability

Supporting tabular data has been provided in supplementary files.

## Code availability

For the present study, we used Python (*scikit* library, open source) and R.
