# Supplementary material for: Machine learning-based analysis of [18F]DCFPyL PET radiomics for risk stratification in primary prostate cancer
Source: Eur J Nucl Med Mol Imaging. 2020 Jul 31;48(2):340–9. doi: 10.1007/s00259-020-04971-z (PMC7835295; doi:10.1007/s00259-020-04971-z)
Supplement: Supplementary file 1 — (PDF 45 kb). [file 259_2020_4971_MOESM1_ESM.pdf]

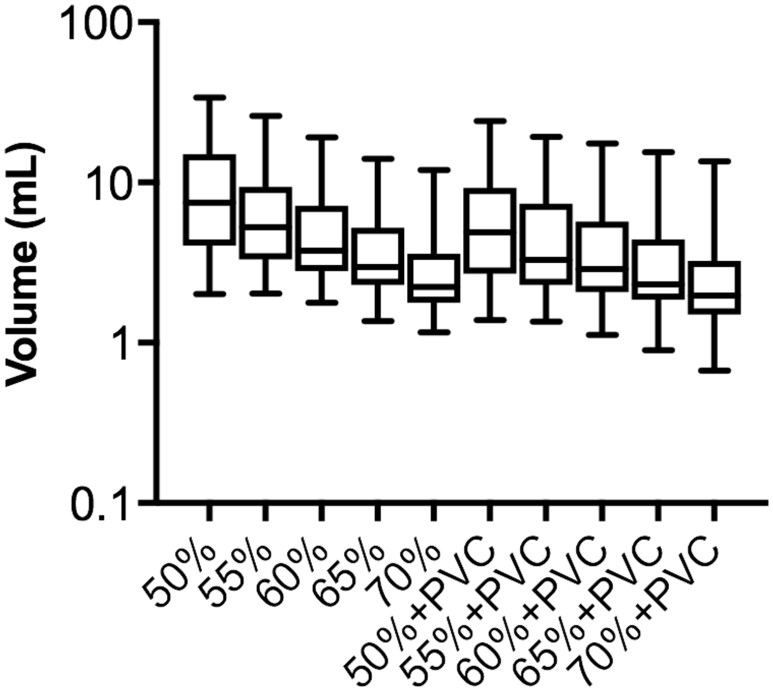

SUPPLEMENTAL FIGURE 1: Delineated PSMA-positive tumor volumes (mL). Data shown for each delineation threshold (peak reference) with and without partial-volume correction (PVC).
