# Supplementary material for: Machine learning-based analysis of [18F]DCFPyL PET radiomics for risk stratification in primary prostate cancer
Source: Eur J Nucl Med Mol Imaging. 2020 Jul 31;48(2):340–9. doi: 10.1007/s00259-020-04971-z (PMC7835295; doi:10.1007/s00259-020-04971-z)
Supplement: Supplementary file 2 — (PDF 570 kb). [file 259_2020_4971_MOESM2_ESM.pdf]

Supplemental Table 1: Radiomics features.

Feature type	Feature name
Morphology	Volume
Morphology	approximate volume
Morphology	Surface
Morphology	Surface to volume ratio
Morphology	Compactness1
Morphology	Compactness2
Morphology	Spherical disproportion
Morphology	sphericity
Morphology	asphericity
Morphology	center of mass shift
Morphology	maximum 3D diameter
Morphology	major axis length
Morphology	minor axis length
Morphology	least axis length
Morphology	elongation
Morphology	flatness
Morphology	vol density AABB
Morphology	area density AABB
Morphology	vol density AEE
Morphology	integrated intensity
Morphology	Morans I
Morphology	Gearys C
Local intensity	local intensity peak
Local intensity	global intensity peak
Intensity statistics	mean
Intensity statistics	variance
Intensity statistics	skewness
Intensity statistics	kurtosis
Intensity statistics	median
Intensity statistics	minimum
Intensity statistics	10th percentile
Intensity statistics	90th percentile
Intensity statistics	maximum
Intensity statistics	Interquartile range
Intensity statistics	range
Intensity statistics	Mean absolut deviation
Intensity statistics	Robust mean absolute deviation
Intensity statistics	Median absolute deviation
Intensity statistics	Coefficient of variation

Intensity statistics	Quartile coefficient
Intensity statistics	Energy
Intensity statistics	Root mean
intensity volume	volume at int fraction 10
intensity volume	volume at int fraction 90
intensity volume	int at vol fraction 10
intensity volume	int at vol fraction 90
intensity volume	difference vol at int fraction
intensity volume	difference int at volume fraction
Intensity histogram	mean
Intensity histogram	variance
Intensity histogram	skewness
Intensity histogram	kurtosis
Intensity histogram	median
Intensity histogram	minimum
Intensity histogram	10th percentile
Intensity histogram	90th percentile
Intensity histogram	maximum
Intensity histogram	mode
Intensity histogram	Interquartile range
Intensity histogram	range
Intensity histogram	Mean absolute deviation
Intensity histogram	Robust mean absolute deviation
Intensity histogram	Median absolute deviation
Intensity histogram	Coefficient of variation
Intensity histogram	Quartile coefficient
Intensity histogram	Entropy
Intensity histogram	Uniformity
Intensity histogram	Energy
Intensity histogram	Maximum histogram gradient
Intensity histogram	Maximum histogram gradient grey level
Intensity histogram	Minimum histogram gradient
Intensity histogram	Minimum histogram gradient grey level
glcmFeatures2Davg	joint maximum
glcmFeatures2Davg	joint average
glcmFeatures2Davg	joint variance
glcmFeatures2Davg	joint entropy
glcmFeatures2Davg	difference average
glcmFeatures2Davg	difference variance
glcmFeatures2Davg	difference entropy
glcmFeatures2Davg	sum average
glcmFeatures2Davg	sum variance

glcmFeatures2Davg	sum entropy
glcmFeatures2Davg	angular second moment
glcmFeatures2Davg	contrast
glcmFeatures2Davg	dissimilarity
glcmFeatures2Davg	inverse difference
glcmFeatures2Davg	inverse difference normalised
glcmFeatures2Davg	inverse difference moment
glcmFeatures2Davg	inverse difference moment normalised
glcmFeatures2Davg	inverse variance
glcmFeatures2Davg	correlation
glcmFeatures2Davg	autocorrelation
glcmFeatures2Davg	cluster tendency
glcmFeatures2Davg	cluster shade
glcmFeatures2Davg	cluster prominence
glcmFeatures2Davg	first measure of information correlation
glcmFeatures2Davg	second measure of information correlation
glcmFeatures2DDmrg	joint maximum
glcmFeatures2DDmrg	joint average
glcmFeatures2DDmrg	joint variance
glcmFeatures2DDmrg	joint entropy
glcmFeatures2DDmrg	difference average
glcmFeatures2DDmrg	difference variance
glcmFeatures2DDmrg	difference entropy
glcmFeatures2DDmrg	sum average
glcmFeatures2DDmrg	sum variance
glcmFeatures2DDmrg	sum entropy
glcmFeatures2DDmrg	angular second moment
glcmFeatures2DDmrg	contrast
glcmFeatures2DDmrg	dissimilarity
glcmFeatures2DDmrg	inverse difference
glcmFeatures2DDmrg	inverse difference normalised
glcmFeatures2DDmrg	inverse difference moment
glcmFeatures2DDmrg	inverse difference moment normalised
glcmFeatures2DDmrg	inverse variance
glcmFeatures2DDmrg	correlation
glcmFeatures2DDmrg	autocorrelation
glcmFeatures2DDmrg	cluster tendency
glcmFeatures2DDmrg	cluster shade
glcmFeatures2DDmrg	cluster prominence
glcmFeatures2DDmrg	first measure of information correlation
glcmFeatures2DDmrg	second measure of information correlation
glcmFeatures2Dmrg	joint maximum

glcmFeatures2Dmrg	joint average
glcmFeatures2Dmrg	joint variance
glcmFeatures2Dmrg	joint entropy
glcmFeatures2Dmrg	difference average
glcmFeatures2Dmrg	difference variance
glcmFeatures2Dmrg	difference entropy
glcmFeatures2Dmrg	sum average
glcmFeatures2Dmrg	sum variance
glcmFeatures2Dmrg	sum entropy
glcmFeatures2Dmrg	angular second moment
glcmFeatures2Dmrg	contrast
glcmFeatures2Dmrg	dissimilarity
glcmFeatures2Dmrg	inverse difference
glcmFeatures2Dmrg	inverse difference normalised
glcmFeatures2Dmrg	inverse difference moment
glcmFeatures2Dmrg	inverse difference moment normalised
glcmFeatures2Dmrg	inverse variance
glcmFeatures2Dmrg	correlation
glcmFeatures2Dmrg	autocorrelation
glcmFeatures2Dmrg	cluster tendency
glcmFeatures2Dmrg	cluster shade
glcmFeatures2Dmrg	cluster prominence
glcmFeatures2Dmrg	first measure of information correlation
glcmFeatures2Dmrg	second measure of information correlation
glcmFeatures2Dvmrg	joint maximum
glcmFeatures2Dvmrg	joint average
glcmFeatures2Dvmrg	joint variance
glcmFeatures2Dvmrg	joint entropy
glcmFeatures2Dvmrg	difference average
glcmFeatures2Dvmrg	difference variance
glcmFeatures2Dvmrg	difference entropy
glcmFeatures2Dvmrg	sum average
glcmFeatures2Dvmrg	sum variance
glcmFeatures2Dvmrg	sum entropy
glcmFeatures2Dvmrg	angular second moment
glcmFeatures2Dvmrg	contrast
glcmFeatures2Dvmrg	dissimilarity
glcmFeatures2Dvmrg	inverse difference
glcmFeatures2Dvmrg	inverse difference normalised
glcmFeatures2Dvmrg	inverse difference moment
glcmFeatures2Dvmrg	inverse difference moment normalised
glcmFeatures2Dvmrg	inverse variance

glcmFeatures2Dvmrg	correlation
glcmFeatures2Dvmrg	autocorrelation
glcmFeatures2Dvmrg	cluster tendency
glcmFeatures2Dvmrg	cluster shade
glcmFeatures2Dvmrg	cluster prominence
glcmFeatures2Dvmrg	first measure of information correlation
glcmFeatures2Dvmrg	second measure of information correlation
glcmFeatures3Davg	joint maximum
glcmFeatures3Davg	joint average
glcmFeatures3Davg	joint variance
glcmFeatures3Davg	joint entropy
glcmFeatures3Davg	difference average
glcmFeatures3Davg	difference variance
glcmFeatures3Davg	difference entropy
glcmFeatures3Davg	sum average
glcmFeatures3Davg	sum variance
glcmFeatures3Davg	sum entropy
glcmFeatures3Davg	angular second moment
glcmFeatures3Davg	contrast
glcmFeatures3Davg	dissimilarity
glcmFeatures3Davg	inverse difference
glcmFeatures3Davg	inverse difference normalised
glcmFeatures3Davg	inverse difference moment
glcmFeatures3Davg	inverse difference moment normalised
glcmFeatures3Davg	inverse variance
glcmFeatures3Davg	correlation
glcmFeatures3Davg	autocorrelation
glcmFeatures3Davg	cluster tendency
glcmFeatures3Davg	cluster shade
glcmFeatures3Davg	cluster prominence
glcmFeatures3Davg	first measure of information correlation
glcmFeatures3Davg	second measure of information correlation
glcmFeatures3DWmrg	joint maximum
glcmFeatures3DWmrg	joint average
glcmFeatures3DWmrg	joint variance
glcmFeatures3DWmrg	joint entropy
glcmFeatures3DWmrg	difference average
glcmFeatures3DWmrg	difference variance
glcmFeatures3DWmrg	difference entropy
glcmFeatures3DWmrg	sum average
glcmFeatures3DWmrg	sum variance
glcmFeatures3DWmrg	sum entropy

glcmFeatures3DWmrg	angular second moment
glcmFeatures3DWmrg	contrast
glcmFeatures3DWmrg	dissimilarity
glcmFeatures3DWmrg	inverse difference
glcmFeatures3DWmrg	inverse difference normalised
glcmFeatures3DWmrg	inverse difference moment
glcmFeatures3DWmrg	inverse difference moment normalised
glcmFeatures3DWmrg	inverse variance
glcmFeatures3DWmrg	correlation
glcmFeatures3DWmrg	autocorrelation
glcmFeatures3DWmrg	cluster tendency
glcmFeatures3DWmrg	cluster shade
glcmFeatures3DWmrg	cluster prominence
glcmFeatures3DWmrg	first measure of information correlation
glcmFeatures3DWmrg	second measure of information correlation
GLRLMFeatures2Davg	short run emphasis
GLRLMFeatures2Davg	long runs emphasis
GLRLMFeatures2Davg	Low grey level run emphasis
GLRLMFeatures2Davg	High grey level run emphasis
GLRLMFeatures2Davg	Short run low grey level emphasis
GLRLMFeatures2Davg	Short run high grey level emphasis
GLRLMFeatures2Davg	Long run low grey level emphasis
GLRLMFeatures2Davg	Long run high grey level emphasis
GLRLMFeatures2Davg	Grey level non uniformity
GLRLMFeatures2Davg	Grey level non uniformity normalized
GLRLMFeatures2Davg	Run length non uniformity
GLRLMFeatures2Davg	Run length non uniformity normalized
GLRLMFeatures2Davg	Run percentage
GLRLMFeatures2Davg	Grey level variance
GLRLMFeatures2Davg	Run length variance
GLRLMFeatures2Davg	Run entropy
GLRLMFeatures2DDmrg	short run emphasis
GLRLMFeatures2DDmrg	long runs emphasis
GLRLMFeatures2DDmrg	Low grey level run emphasis
GLRLMFeatures2DDmrg	High grey level run emphasis
GLRLMFeatures2DDmrg	Short run low grey level emphasis
GLRLMFeatures2DDmrg	Short run high grey level emphasis
GLRLMFeatures2DDmrg	Long run low grey level emphasis
GLRLMFeatures2DDmrg	Long run high grey level emphasis
GLRLMFeatures2DDmrg	Grey level non uniformity
GLRLMFeatures2DDmrg	Grey level non uniformity normalized
GLRLMFeatures2DDmrg	Run length non uniformity

GLRLMFeatures2DDmrg	Run length non uniformity normalized
GLRLMFeatures2DDmrg	Run percentage
GLRLMFeatures2DDmrg	Grey level variance
GLRLMFeatures2DDmrg	Run length variance
GLRLMFeatures2DDmrg	Run entropy
GLRLMFeatures2DWmrg	short run emphasis
GLRLMFeatures2DWmrg	long runs emphasis
GLRLMFeatures2DWmrg	Low grey level run emphasis
GLRLMFeatures2DWmrg	High grey level run emphasis
GLRLMFeatures2DWmrg	Short run low grey level emphasis
GLRLMFeatures2DWmrg	Short run high grey level emphasis
GLRLMFeatures2DWmrg	Long run low grey level emphasis
GLRLMFeatures2DWmrg	Long run high grey level emphasis
GLRLMFeatures2DWmrg	Grey level non uniformity
GLRLMFeatures2DWmrg	Grey level non uniformity normalized
GLRLMFeatures2DWmrg	Run length non uniformity
GLRLMFeatures2DWmrg	Run length non uniformity normalized
GLRLMFeatures2DWmrg	Run percentage
GLRLMFeatures2DWmrg	Grey level variance
GLRLMFeatures2DWmrg	Run length variance
GLRLMFeatures2DWmrg	Run entropy
GLRLMFeatures2Dvmrg	short run emphasis
GLRLMFeatures2Dvmrg	long runs emphasis
GLRLMFeatures2Dvmrg	Low grey level run emphasis
GLRLMFeatures2Dvmrg	High grey level run emphasis
GLRLMFeatures2Dvmrg	Short run low grey level emphasis
GLRLMFeatures2Dvmrg	Short run high grey level emphasis
GLRLMFeatures2Dvmrg	Long run low grey level emphasis
GLRLMFeatures2Dvmrg	Long run high grey level emphasis
GLRLMFeatures2Dvmrg	Grey level non uniformity
GLRLMFeatures2Dvmrg	Grey level non uniformity normalized
GLRLMFeatures2Dvmrg	Run length non uniformity
GLRLMFeatures2Dvmrg	Run length non uniformity normalized
GLRLMFeatures2Dvmrg	Run percentage
GLRLMFeatures2Dvmrg	Grey level variance
GLRLMFeatures2Dvmrg	Run length variance
GLRLMFeatures2Dvmrg	Run entropy
GLRLMFeatures3Davg	short run emphasis
GLRLMFeatures3Davg	long runs emphasis
GLRLMFeatures3Davg	Low grey level run emphasis
GLRLMFeatures3Davg	High grey level run emphasis
GLRLMFeatures3Davg	Short run low grey level emphasis

GLRLMFeatures3Davg	Short run high grey level emphasis
GLRLMFeatures3Davg	Long run low grey level emphasis
GLRLMFeatures3Davg	Long run high grey level emphasis
GLRLMFeatures3Davg	Grey level non uniformity
GLRLMFeatures3Davg	Grey level non uniformity normalized
GLRLMFeatures3Davg	Run length non uniformity
GLRLMFeatures3Davg	Run length non uniformity normalized
GLRLMFeatures3Davg	Run percentage
GLRLMFeatures3Davg	Grey level variance
GLRLMFeatures3Davg	Run length variance
GLRLMFeatures3Davg	Run entropy
GLRLMFeatures3Dmrg	short run emphasis
GLRLMFeatures3Dmrg	long runs emphasis
GLRLMFeatures3Dmrg	Low grey level run emphasis
GLRLMFeatures3Dmrg	High grey level run emphasis
GLRLMFeatures3Dmrg	Short run low grey level emphasis
GLRLMFeatures3Dmrg	Short run high grey level emphasis
GLRLMFeatures3Dmrg	Long run low grey level emphasis
GLRLMFeatures3Dmrg	Long run high grey level emphasis
GLRLMFeatures3Dmrg	Grey level non uniformity
GLRLMFeatures3Dmrg	Grey level non uniformity normalized
GLRLMFeatures3Dmrg	Run length non uniformity
GLRLMFeatures3Dmrg	Run length non uniformity normalized
GLRLMFeatures3Dmrg	Run percentage
GLRLMFeatures3Dmrg	Grey level variance
GLRLMFeatures3Dmrg	Run length variance
GLRLMFeatures3Dmrg	Run entropy
GLSZMFeatures2Davg	small zone emphasis
GLSZMFeatures2Davg	Large zone emphasis
GLSZMFeatures2Davg	Low grey level zone emphasis
GLSZMFeatures2Davg	High grey level zone emphasis
GLSZMFeatures2Davg	Small zone low grey level emphasis
GLSZMFeatures2Davg	Small zone high grey level emphasis
GLSZMFeatures2Davg	Large zone low grey level emphasis
GLSZMFeatures2Davg	Large zone high grey level emphasis
GLSZMFeatures2Davg	Grey level non uniformity GLSZM
GLSZMFeatures2Davg	Grey level non uniformity normalized GLSZM
GLSZMFeatures2Davg	Zone size non uniformity
GLSZMFeatures2Davg	Zone size non uniformity normalized
GLSZMFeatures2Davg	Zone percentage GLSZM
GLSZMFeatures2Davg	Grey level variance GLSZM
GLSZMFeatures2Davg	Zone size variance

GLSZMFeatures2Davg	Zone size entropy
GLSZMFeatures2Dvmrg	small zone emphasis
GLSZMFeatures2Dvmrg	Large zone emphasis
GLSZMFeatures2Dvmrg	Low grey level zone emphasis
GLSZMFeatures2Dvmrg	High grey level zone emphasis
GLSZMFeatures2Dvmrg	Small zone low grey level emphasis
GLSZMFeatures2Dvmrg	Small zone high grey level emphasis
GLSZMFeatures2Dvmrg	Large zone low grey level emphasis
GLSZMFeatures2Dvmrg	Large zone high grey level emphasis
GLSZMFeatures2Dvmrg	Grey level non uniformity GLSZM
GLSZMFeatures2Dvmrg	Grey level non uniformity normalized GLSZM
GLSZMFeatures2Dvmrg	Zone size non uniformity
GLSZMFeatures2Dvmrg	Zone size non uniformity normalized
GLSZMFeatures2Dvmrg	Zone percentage GLSZM
GLSZMFeatures2Dvmrg	Grey level variance GLSZM
GLSZMFeatures2Dvmrg	Zone size variance
GLSZMFeatures2Dvmrg	Zone size entropy
GLSZMFeatures3D	small zone emphasis
GLSZMFeatures3D	Large zone emphasis
GLSZMFeatures3D	Low grey level zone emphasis
GLSZMFeatures3D	High grey level zone emphasis
GLSZMFeatures3D	Small zone low grey level emphasis
GLSZMFeatures3D	Small zone high grey level emphasis
GLSZMFeatures3D	Large zone low grey level emphasis
GLSZMFeatures3D	Large zone high grey level emphasis
GLSZMFeatures3D	Grey level non uniformity GLSZM
GLSZMFeatures3D	Grey level non uniformity normalized GLSZM
GLSZMFeatures3D	Zone size non uniformity
GLSZMFeatures3D	Zone size non uniformity normalized
GLSZMFeatures3D	Zone percentage GLSZM
GLSZMFeatures3D	Grey level variance GLSZM
GLSZMFeatures3D	Zone size variance
GLSZMFeatures3D	Zone size entropy
ngtdmFeatures2avg	coarseness
ngtdmFeatures2avg	contrast
ngtdmFeatures2avg	busyness
ngtdmFeatures2avg	complexity
ngtdmFeatures2avg	strength
ngtdmFeatures2Dmrg	coarseness
ngtdmFeatures2Dmrg	contrast
ngtdmFeatures2Dmrg	busyness
ngtdmFeatures2Dmrg	complexity

ngtdmFeatures2Dmrg	strength
ngtdmFeatures3D	coarseness
ngtdmFeatures3D	contrast
ngtdmFeatures3D	busyness
ngtdmFeatures3D	complexity
ngtdmFeatures3D	strength
gldzmFeatures2Davg	small distance emphasis GLDZM
gldzmFeatures2Davg	Large distance emphasis GLDZM
gldzmFeatures2Davg	Low grey level zone emphasis GLDZM
gldzmFeatures2Davg	High grey level zone emphasis GLDZM
gldzmFeatures2Davg	Small distance low grey level emphasis GLDZM
gldzmFeatures2Davg	Small distance high grey level emphasis GLDZM
gldzmFeatures2Davg	Large distance low grey level emphasis GLDZM
gldzmFeatures2Davg	Large distance high grey level emphasis GLDZM
gldzmFeatures2Davg	Grey level non uniformity GLDZM
gldzmFeatures2Davg	Grey level non uniformity normalized GLDZM
gldzmFeatures2Davg	Zone distance non uniformity GLDZM
gldzmFeatures2Davg	Zone distance non uniformity normalized GLDZM
gldzmFeatures2Davg	Zone percentage GLDZM
gldzmFeatures2Davg	Grey level variance GLDZM
gldzmFeatures2Davg	Zone distance variance GLDZM
gldzmFeatures2Davg	Zone distance entropy GLDZM
gldzmFeatures2Dmrg	small distance emphasis GLDZM
gldzmFeatures2Dmrg	Large distance emphasis GLDZM
gldzmFeatures2Dmrg	Low grey level zone emphasis GLDZM
gldzmFeatures2Dmrg	High grey level zone emphasis GLDZM
gldzmFeatures2Dmrg	Small distance low grey level emphasis GLDZM
gldzmFeatures2Dmrg	Small distance high grey level emphasis GLDZM
gldzmFeatures2Dmrg	Large distance low grey level emphasis GLDZM
gldzmFeatures2Dmrg	Large distance high grey level emphasis GLDZM
gldzmFeatures2Dmrg	Grey level non uniformity GLDZM
gldzmFeatures2Dmrg	Grey level non uniformity normalized GLDZM
gldzmFeatures2Dmrg	Zone distance non uniformity GLDZM
gldzmFeatures2Dmrg	Zone distance non uniformity normalized GLDZM
gldzmFeatures2Dmrg	Zone percentage GLDZM
gldzmFeatures2Dmrg	Grey level variance GLDZM
gldzmFeatures2Dmrg	Zone distance variance GLDZM

gldzmFeatures2Dmrg	Zone distance entropy GLDZM
gldzmFeatures3D	small distance emphasis GLDZM
gldzmFeatures3D	Large distance emphasis GLDZM
gldzmFeatures3D	Low grey level zone emphasis GLDZM
gldzmFeatures3D	High grey level zone emphasis GLDZM
gldzmFeatures3D	Small distance low grey level emphasis GLDZM
gldzmFeatures3D	Small distance high grey level emphasis GLDZM
gldzmFeatures3D	Large distance low grey level emphasis GLDZM
gldzmFeatures3D	Large distance high grey level emphasis GLDZM
gldzmFeatures3D	Grey level non uniformity GLDZM
gldzmFeatures3D	Grey level non uniformity normalized GLDZM
gldzmFeatures3D	Zone distance non uniformity GLDZM
gldzmFeatures3D	Zone distance non uniformity normalized GLDZM
gldzmFeatures3D	Zone percentage GLDZM
gldzmFeatures3D	Grey level variance GLDZM
gldzmFeatures3D	Zone distance variance GLDZM
gldzmFeatures3D	Zone distance entropy GLDZM
ngldmFeatures2Davg	Low dependence emphasis
ngldmFeatures2Davg	High dependence emphasis
ngldmFeatures2Davg	Low grey level count emphasis
ngldmFeatures2Davg	High grey level count emphasis
ngldmFeatures2Davg	Low dependence low grey level emphasis
ngldmFeatures2Davg	Low dependence high grey level emphasis
ngldmFeatures2Davg	High dependence low grey level emphasis
ngldmFeatures2Davg	High dependence high grey level emphasis
ngldmFeatures2Davg	Grey level non uniformity
ngldmFeatures2Davg	Grey level non uniformity normalized
ngldmFeatures2Davg	Dependence count non uniformity
ngldmFeatures2Davg	Dependence count non uniformity normalized
ngldmFeatures2Davg	Dependence count percentage
ngldmFeatures2Davg	Grey level variance
ngldmFeatures2Davg	Dependence count variance
ngldmFeatures2Davg	Dependence count entropy
ngldmFeatures2Davg	dependence Count Energy
ngldmFeatures2Dmrg	Low dependence emphasis
ngldmFeatures2Dmrg	High dependence emphasis
ngldmFeatures2Dmrg	Low grey level count emphasis
ngldmFeatures2Dmrg	High grey level count emphasis
ngldmFeatures2Dmrg	Low dependence low grey level emphasis
ngldmFeatures2Dmrg	Low dependence high grey level emphasis

ngldmFeatures2Dmrg	High dependence low grey level emphasis
ngldmFeatures2Dmrg	High dependence high grey level emphasis
ngldmFeatures2Dmrg	Grey level non uniformity
ngldmFeatures2Dmrg	Grey level non uniformity normalized
ngldmFeatures2Dmrg	Dependence count non uniformity
ngldmFeatures2Dmrg	Dependence count non uniformity normalized
ngldmFeatures2Dmrg	Dependence count percentage
ngldmFeatures2Dmrg	Grey level variance
ngldmFeatures2Dmrg	Dependence count variance
ngldmFeatures2Dmrg	Dependence count entropy
ngldmFeatures2Dmrg	dependence Count Energy
ngldmFeatures3Dmrg	Low dependence emphasis
ngldmFeatures3Dmrg	High dependence emphasis
ngldmFeatures3Dmrg	Low grey level count emphasis
ngldmFeatures3Dmrg	High grey level count emphasis
ngldmFeatures3Dmrg	Low dependence low grey level emphasis
ngldmFeatures3Dmrg	Low dependence high grey level emphasis
ngldmFeatures3Dmrg	High dependence low grey level emphasis
ngldmFeatures3Dmrg	High dependence high grey level emphasis
ngldmFeatures3Dmrg	Grey level non uniformity
ngldmFeatures3Dmrg	Grey level non uniformity normalized
ngldmFeatures3Dmrg	Dependence count non uniformity
ngldmFeatures3Dmrg	Dependence count non uniformity normalized
ngldmFeatures3Dmrg	Dependence count percentage
ngldmFeatures3Dmrg	Grey level variance
ngldmFeatures3Dmrg	Dependence count variance
ngldmFeatures3Dmrg	Dependence count entropy
ngldmFeatures3Dmrg	dependence Count Energy
