# Supplementary material for: Machine learning-based analysis of [18F]DCFPyL PET radiomics for risk stratification in primary prostate cancer
Source: Eur J Nucl Med Mol Imaging. 2020 Jul 31;48(2):340–9. doi: 10.1007/s00259-020-04971-z (PMC7835295; doi:10.1007/s00259-020-04971-z)
Supplement: Supplementary file 3 — (PDF 135 kb). [file 259_2020_4971_MOESM3_ESM.pdf]

Supplementary Table 2: Results from all cross-validation analyses.

Outcome	Partial-volume correction	Delineation threshold	Feature selection	Oversampling	AUC mean	AUC std	Brier score mean	Brier score std
LNI	No	50%	None	None	0.70	0.24	0.10	0.04
LNI	No	50%	PCA	None	0.65	0.24	0.11	0.03
LNI	No	50%	RFE-RF	None	0.67	0.25	0.10	0.04
LNI	No	50%	Univariate	None	0.65	0.24	0.10	0.04
LNI	No	50%	None	SMOTE	0.70	0.23	0.12	0.04
LNI	No	50%	PCA	SMOTE	0.66	0.23	0.13	0.04
LNI	No	50%	RFE-RF	SMOTE	0.68	0.22	0.14	0.05
LNI	No	50%	Univariate	SMOTE	0.69	0.22	0.15	0.05
LNI	No	55%	None	None	0.71	0.23	0.10	0.04
LNI	No	55%	PCA	None	0.77	0.18	0.10	0.03
LNI	No	55%	RFE-RF	None	0.68	0.24	0.11	0.04
LNI	No	55%	Univariate	None	0.63	0.23	0.11	0.04
LNI	No	55%	None	SMOTE	0.70	0.25	0.13	0.05
LNI	No	55%	PCA	SMOTE	0.71	0.20	0.13	0.04
LNI	No	55%	RFE-RF	SMOTE	0.65	0.25	0.14	0.05
LNI	No	55%	Univariate	SMOTE	0.62	0.24	0.16	0.05
LNI	No	60%	None	None	0.73	0.22	0.10	0.04
LNI	No	60%	PCA	None	0.71	0.21	0.11	0.03
LNI	No	60%	RFE-RF	None	0.65	0.24	0.11	0.05
LNI	No	60%	Univariate	None	0.61	0.25	0.11	0.04
LNI	No	60%	None	SMOTE	0.72	0.24	0.12	0.05
LNI	No	60%	PCA	SMOTE	0.68	0.20	0.14	0.04
LNI	No	60%	RFE-RF	SMOTE	0.68	0.23	0.14	0.06
LNI	No	60%	Univariate	SMOTE	0.62	0.26	0.16	0.05
LNI	No	65%	None	None	0.69	0.23	0.11	0.04
LNI	No	65%	PCA	None	0.62	0.22	0.12	0.03
LNI	No	65%	RFE-RF	None	0.64	0.23	0.12	0.04
LNI	No	65%	Univariate	None	0.59	0.23	0.12	0.04
LNI	No	65%	None	SMOTE	0.70	0.24	0.13	0.04
LNI	No	65%	PCA	SMOTE	0.64	0.21	0.15	0.04
LNI	No	65%	RFE-RF	SMOTE	0.64	0.23	0.15	0.05
LNI	No	65%	Univariate	SMOTE	0.64	0.23	0.16	0.05
LNI	No	70%	None	None	0.70	0.22	0.10	0.04
LNI	No	70%	PCA	None	0.73	0.19	0.11	0.03
LNI	No	70%	RFE-RF	None	0.65	0.23	0.11	0.04
LNI	No	70%	Univariate	None	0.67	0.21	0.11	0.04
LNI	No	70%	None	SMOTE	0.73	0.22	0.12	0.04
LNI	No	70%	PCA	SMOTE	0.74	0.18	0.12	0.04
LNI	No	70%	RFE-RF	SMOTE	0.69	0.24	0.13	0.05
LNI	No	70%	Univariate	SMOTE	0.72	0.20	0.15	0.05
LNI	Yes	50%	None	None	0.74	0.21	0.09	0.03
LNI	Yes	50%	PCA	None	0.74	0.20	0.10	0.03
LNI	Yes	50%	RFE-RF	None	0.71	0.21	0.10	0.04
LNI	Yes	50%	Univariate	None	0.67	0.23	0.10	0.04
LNI	Yes	50%	None	SMOTE	0.74	0.22	0.12	0.04
LNI	Yes	50%	PCA	SMOTE	0.80	0.16	0.12	0.04
LNI	Yes	50%	RFE-RF	SMOTE	0.71	0.22	0.13	0.05
LNI	Yes	50%	Univariate	SMOTE	0.68	0.24	0.15	0.05
LNI	Yes	55%	None	None	0.74	0.19	0.10	0.04
LNI	Yes	55%	PCA	None	0.73	0.19	0.11	0.03
LNI	Yes	55%	RFE-RF	None	0.73	0.20	0.10	0.04

LNI	Yes	55%	Univariate	None	0.66	0.23	0.11	0.04
LNI	Yes	55%	None	SMOTE	0.75	0.22	0.11	0.05
LNI	Yes	55%	PCA	SMOTE	0.75	0.16	0.13	0.04
LNI	Yes	55%	RFE-RF	SMOTE	0.74	0.21	0.12	0.05
LNI	Yes	55%	Univariate	SMOTE	0.71	0.23	0.14	0.06
LNI	Yes	60%	None	None	0.76	0.16	0.10	0.04
LNI	Yes	60%	PCA	None	0.77	0.17	0.10	0.03
LNI	Yes	60%	RFE-RF	None	0.75	0.18	0.10	0.04
LNI	Yes	60%	Univariate	None	0.71	0.18	0.10	0.04
LNI	Yes	60%	None	SMOTE	0.74	0.19	0.12	0.04
LNI	Yes	60%	PCA	SMOTE	0.79	0.15	0.12	0.04
LNI	Yes	60%	RFE-RF	SMOTE	0.74	0.19	0.13	0.05
LNI	Yes	60%	Univariate	SMOTE	0.73	0.19	0.14	0.05
LNI	Yes	65%	None	None	0.80	0.15	0.10	0.03
LNI	Yes	65%	PCA	None	0.78	0.16	0.10	0.03
LNI	Yes	65%	RFE-RF	None	0.79	0.15	0.10	0.04
LNI	Yes	65%	Univariate	None	0.76	0.17	0.10	0.04
LNI	Yes	65%	None	SMOTE	0.81	0.17	0.11	0.04
LNI	Yes	65%	PCA	SMOTE	0.81	0.13	0.12	0.04
LNI	Yes	65%	RFE-RF	SMOTE	0.81	0.16	0.11	0.05
LNI	Yes	65%	Univariate	SMOTE	0.79	0.17	0.12	0.05
LNI	Yes	70%	None	None	0.81	0.15	0.09	0.03
LNI	Yes	70%	PCA	None	0.81	0.14	0.10	0.02
LNI	Yes	70%	RFE-RF	None	0.79	0.16	0.10	0.04
LNI	Yes	70%	Univariate	None	0.84	0.15	0.09	0.04
LNI	Yes	70%	None	SMOTE	0.84	0.16	0.10	0.04
LNI	Yes	70%	PCA	SMOTE	0.85	0.13	0.10	0.04
LNI	Yes	70%	RFE-RF	SMOTE	0.85	0.14	0.09	0.04
LNI	Yes	70%	Univariate	SMOTE	0.86	0.15	0.09	0.05
Any metastasis	No	50%	None	None	0.78	0.18	0.10	0.04
Any metastasis	No	50%	PCA	None	0.75	0.19	0.11	0.04
Any metastasis	No	50%	RFE-RF	None	0.76	0.19	0.10	0.04
Any metastasis	No	50%	Univariate	None	0.75	0.20	0.10	0.04
Any metastasis	No	50%	None	SMOTE	0.79	0.18	0.12	0.04
Any metastasis	No	50%	PCA	SMOTE	0.75	0.17	0.13	0.04
Any metastasis	No	50%	RFE-RF	SMOTE	0.77	0.18	0.13	0.05
Any metastasis	No	50%	Univariate	SMOTE	0.78	0.17	0.13	0.05
Any metastasis	No	55%	None	None	0.79	0.18	0.10	0.04
Any metastasis	No	55%	PCA	None	0.83	0.15	0.11	0.03
Any metastasis	No	55%	RFE-RF	None	0.77	0.20	0.10	0.04
Any metastasis	No	55%	Univariate	None	0.75	0.20	0.10	0.04
Any metastasis	No	55%	None	SMOTE	0.79	0.19	0.12	0.04
Any metastasis	No	55%	PCA	SMOTE	0.82	0.15	0.12	0.04
Any metastasis	No	55%	RFE-RF	SMOTE	0.76	0.20	0.13	0.05
Any metastasis	No	55%	Univariate	SMOTE	0.75	0.20	0.13	0.05
Any metastasis	No	60%	None	None	0.79	0.17	0.11	0.04
Any metastasis	No	60%	PCA	None	0.77	0.16	0.12	0.04
Any metastasis	No	60%	RFE-RF	None	0.74	0.20	0.11	0.05
Any metastasis	No	60%	Univariate	None	0.71	0.21	0.11	0.04
Any metastasis	No	60%	None	SMOTE	0.80	0.18	0.12	0.04
Any metastasis	No	60%	PCA	SMOTE	0.78	0.15	0.14	0.04
Any metastasis	No	60%	RFE-RF	SMOTE	0.77	0.19	0.13	0.05
Any metastasis	No	60%	Univariate	SMOTE	0.75	0.20	0.14	0.05
Any metastasis	No	65%	None	None	0.75	0.18	0.12	0.04
Any metastasis	No	65%	PCA	None	0.74	0.17	0.12	0.04
Any metastasis	No	65%	RFE-RF	None	0.71	0.19	0.12	0.04
Any metastasis	No	65%	Univariate	None	0.72	0.18	0.12	0.04
Any metastasis	No	65%	None	SMOTE	0.76	0.18	0.13	0.05

Any metastasis	No	65%	PCA	SMOTE	0.76	0.16	0.14	0.04
Any metastasis	No	65%	RFE-RF	SMOTE	0.74	0.18	0.14	0.05
Any metastasis	No	65%	Univariate	SMOTE	0.76	0.18	0.14	0.05
Any metastasis	No	70%	None	None	0.73	0.18	0.12	0.04
Any metastasis	No	70%	PCA	None	0.69	0.17	0.14	0.03
Any metastasis	No	70%	RFE-RF	None	0.69	0.20	0.13	0.05
Any metastasis	No	70%	Univariate	None	0.72	0.17	0.13	0.05
Any metastasis	No	70%	None	SMOTE	0.76	0.17	0.14	0.05
Any metastasis	No	70%	PCA	SMOTE	0.70	0.15	0.16	0.04
Any metastasis	No	70%	RFE-RF	SMOTE	0.75	0.18	0.15	0.05
Any metastasis	No	70%	Univariate	SMOTE	0.77	0.16	0.15	0.05
Any metastasis	Yes	50%	None	None	0.79	0.17	0.10	0.04
Any metastasis	Yes	50%	PCA	None	0.81	0.15	0.11	0.04
Any metastasis	Yes	50%	RFE-RF	None	0.78	0.19	0.10	0.04
Any metastasis	Yes	50%	Univariate	None	0.76	0.19	0.10	0.04
Any metastasis	Yes	50%	None	SMOTE	0.81	0.18	0.11	0.04
Any metastasis	Yes	50%	PCA	SMOTE	0.83	0.13	0.12	0.05
Any metastasis	Yes	50%	RFE-RF	SMOTE	0.78	0.19	0.12	0.05
Any metastasis	Yes	50%	Univariate	SMOTE	0.77	0.19	0.13	0.05
Any metastasis	Yes	55%	None	None	0.80	0.15	0.11	0.04
Any metastasis	Yes	55%	PCA	None	0.78	0.15	0.12	0.04
Any metastasis	Yes	55%	RFE-RF	None	0.79	0.16	0.11	0.04
Any metastasis	Yes	55%	Univariate	None	0.75	0.19	0.11	0.04
Any metastasis	Yes	55%	None	SMOTE	0.81	0.16	0.12	0.04
Any metastasis	Yes	55%	PCA	SMOTE	0.79	0.14	0.14	0.04
Any metastasis	Yes	55%	RFE-RF	SMOTE	0.80	0.16	0.12	0.04
Any metastasis	Yes	55%	Univariate	SMOTE	0.78	0.18	0.13	0.05
Any metastasis	Yes	60%	None	None	0.82	0.14	0.11	0.04
Any metastasis	Yes	60%	PCA	None	0.82	0.14	0.12	0.03
Any metastasis	Yes	60%	RFE-RF	None	0.82	0.15	0.10	0.04
Any metastasis	Yes	60%	Univariate	None	0.80	0.15	0.11	0.04
Any metastasis	Yes	60%	None	SMOTE	0.82	0.15	0.12	0.04
Any metastasis	Yes	60%	PCA	SMOTE	0.84	0.12	0.12	0.04
Any metastasis	Yes	60%	RFE-RF	SMOTE	0.82	0.15	0.12	0.05
Any metastasis	Yes	60%	Univariate	SMOTE	0.82	0.14	0.12	0.05
Any metastasis	Yes	65%	None	None	0.81	0.13	0.11	0.04
Any metastasis	Yes	65%	PCA	None	0.80	0.13	0.12	0.03
Any metastasis	Yes	65%	RFE-RF	None	0.80	0.14	0.12	0.04
Any metastasis	Yes	65%	Univariate	None	0.80	0.14	0.12	0.04
Any metastasis	Yes	65%	None	SMOTE	0.83	0.13	0.12	0.04
Any metastasis	Yes	65%	PCA	SMOTE	0.83	0.12	0.13	0.04
Any metastasis	Yes	65%	RFE-RF	SMOTE	0.82	0.13	0.12	0.05
Any metastasis	Yes	65%	Univariate	SMOTE	0.82	0.13	0.13	0.05
Any metastasis	Yes	70%	None	None	0.77	0.17	0.12	0.04
Any metastasis	Yes	70%	PCA	None	0.76	0.16	0.12	0.03
Any metastasis	Yes	70%	RFE-RF	None	0.79	0.17	0.11	0.04
Any metastasis	Yes	70%	Univariate	None	0.82	0.15	0.10	0.04
Any metastasis	Yes	70%	None	SMOTE	0.82	0.15	0.11	0.04
Any metastasis	Yes	70%	PCA	SMOTE	0.78	0.16	0.13	0.04
Any metastasis	Yes	70%	RFE-RF	SMOTE	0.84	0.15	0.11	0.04
Any metastasis	Yes	70%	Univariate	SMOTE	0.86	0.14	0.10	0.04
Gleason score	No	50%	None	None	0.70	0.16	0.17	0.05
Gleason score	No	50%	PCA	None	0.70	0.14	0.18	0.04
Gleason score	No	50%	RFE-RF	None	0.71	0.16	0.17	0.05
Gleason score	No	50%	Univariate	None	0.71	0.15	0.18	0.05
Gleason score	No	50%	None	SMOTE	0.72	0.17	0.18	0.05
Gleason score	No	50%	PCA	SMOTE	0.69	0.14	0.19	0.05
Gleason score	No	50%	RFE-RF	SMOTE	0.72	0.17	0.18	0.05

Gleason score	No	50%	Univariate	SMOTE	0.70	0.16	0.19	0.05
Gleason score	No	55%	None	None	0.76	0.15	0.16	0.05
Gleason score	No	55%	PCA	None	0.73	0.15	0.17	0.04
Gleason score	No	55%	RFE-RF	None	0.78	0.14	0.15	0.05
Gleason score	No	55%	Univariate	None	0.75	0.15	0.16	0.05
Gleason score	No	55%	None	SMOTE	0.77	0.16	0.16	0.05
Gleason score	No	55%	PCA	SMOTE	0.71	0.15	0.19	0.05
Gleason score	No	55%	RFE-RF	SMOTE	0.78	0.15	0.16	0.05
Gleason score	No	55%	Univariate	SMOTE	0.73	0.16	0.18	0.05
Gleason score	No	60%	None	None	0.76	0.16	0.15	0.05
Gleason score	No	60%	PCA	None	0.69	0.16	0.18	0.04
Gleason score	No	60%	RFE-RF	None	0.77	0.15	0.15	0.05
Gleason score	No	60%	Univariate	None	0.74	0.16	0.15	0.05
Gleason score	No	60%	None	SMOTE	0.76	0.17	0.16	0.05
Gleason score	No	60%	PCA	SMOTE	0.67	0.16	0.19	0.04
Gleason score	No	60%	RFE-RF	SMOTE	0.76	0.16	0.16	0.05
Gleason score	No	60%	Univariate	SMOTE	0.74	0.17	0.17	0.05
Gleason score	No	65%	None	None	0.78	0.16	0.15	0.05
Gleason score	No	65%	PCA	None	0.68	0.16	0.18	0.04
Gleason score	No	65%	RFE-RF	None	0.78	0.15	0.15	0.05
Gleason score	No	65%	Univariate	None	0.74	0.16	0.16	0.05
Gleason score	No	65%	None	SMOTE	0.77	0.16	0.15	0.05
Gleason score	No	65%	PCA	SMOTE	0.67	0.17	0.19	0.05
Gleason score	No	65%	RFE-RF	SMOTE	0.77	0.15	0.16	0.05
Gleason score	No	65%	Univariate	SMOTE	0.74	0.17	0.18	0.06
Gleason score	No	70%	None	None	0.70	0.18	0.17	0.05
Gleason score	No	70%	PCA	None	0.61	0.17	0.20	0.04
Gleason score	No	70%	RFE-RF	None	0.68	0.18	0.18	0.06
Gleason score	No	70%	Univariate	None	0.65	0.19	0.18	0.06
Gleason score	No	70%	None	SMOTE	0.70	0.18	0.18	0.05
Gleason score	No	70%	PCA	SMOTE	0.62	0.16	0.21	0.05
Gleason score	No	70%	RFE-RF	SMOTE	0.68	0.17	0.20	0.05
Gleason score	No	70%	Univariate	SMOTE	0.65	0.19	0.21	0.05
Gleason score	Yes	50%	None	None	0.76	0.16	0.15	0.05
Gleason score	Yes	50%	PCA	None	0.68	0.17	0.18	0.04
Gleason score	Yes	50%	RFE-RF	None	0.78	0.15	0.15	0.05
Gleason score	Yes	50%	Univariate	None	0.74	0.16	0.16	0.05
Gleason score	Yes	50%	None	SMOTE	0.75	0.16	0.16	0.05
Gleason score	Yes	50%	PCA	SMOTE	0.70	0.16	0.19	0.04
Gleason score	Yes	50%	RFE-RF	SMOTE	0.76	0.16	0.16	0.05
Gleason score	Yes	50%	Univariate	SMOTE	0.73	0.17	0.17	0.05
Gleason score	Yes	55%	None	None	0.77	0.15	0.16	0.05
Gleason score	Yes	55%	PCA	None	0.71	0.16	0.17	0.04
Gleason score	Yes	55%	RFE-RF	None	0.79	0.14	0.15	0.05
Gleason score	Yes	55%	Univariate	None	0.76	0.16	0.16	0.05
Gleason score	Yes	55%	None	SMOTE	0.75	0.17	0.17	0.05
Gleason score	Yes	55%	PCA	SMOTE	0.71	0.17	0.18	0.04
Gleason score	Yes	55%	RFE-RF	SMOTE	0.77	0.15	0.17	0.05
Gleason score	Yes	55%	Univariate	SMOTE	0.74	0.17	0.18	0.05
Gleason score	Yes	60%	None	None	0.76	0.16	0.16	0.05
Gleason score	Yes	60%	PCA	None	0.72	0.15	0.17	0.04
Gleason score	Yes	60%	RFE-RF	None	0.77	0.16	0.16	0.06
Gleason score	Yes	60%	Univariate	None	0.80	0.16	0.15	0.06
Gleason score	Yes	60%	None	SMOTE	0.78	0.16	0.16	0.05
Gleason score	Yes	60%	PCA	SMOTE	0.70	0.16	0.18	0.04
Gleason score	Yes	60%	RFE-RF	SMOTE	0.78	0.16	0.16	0.05
Gleason score	Yes	60%	Univariate	SMOTE	0.81	0.16	0.15	0.06
Gleason score	Yes	65%	None	None	0.75	0.16	0.16	0.05

Gleason score	Yes	65%	PCA	None	0.67	0.17	0.18	0.04
Gleason score	Yes	65%	RFE-RF	None	0.72	0.18	0.17	0.06
Gleason score	Yes	65%	Univariate	None	0.72	0.17	0.16	0.06
Gleason score	Yes	65%	None	SMOTE	0.77	0.16	0.16	0.05
Gleason score	Yes	65%	PCA	SMOTE	0.66	0.16	0.20	0.04
Gleason score	Yes	65%	RFE-RF	SMOTE	0.75	0.17	0.17	0.06
Gleason score	Yes	65%	Univariate	SMOTE	0.72	0.17	0.17	0.05
Gleason score	Yes	70%	None	None	0.70	0.18	0.17	0.05
Gleason score	Yes	70%	PCA	None	0.67	0.18	0.18	0.04
Gleason score	Yes	70%	RFE-RF	None	0.72	0.17	0.16	0.05
Gleason score	Yes	70%	Univariate	None	0.70	0.18	0.16	0.05
Gleason score	Yes	70%	None	SMOTE	0.75	0.18	0.16	0.05
Gleason score	Yes	70%	PCA	SMOTE	0.67	0.17	0.20	0.04
Gleason score	Yes	70%	RFE-RF	SMOTE	0.74	0.17	0.17	0.05
Gleason score	Yes	70%	Univariate	SMOTE	0.74	0.16	0.17	0.05
ECE	No	50%	None	None	0.63	0.13	0.24	0.04
ECE	No	50%	PCA	None	0.60	0.14	0.25	0.04
ECE	No	50%	RFE-RF	None	0.62	0.13	0.25	0.05
ECE	No	50%	Univariate	None	0.62	0.14	0.25	0.05
ECE	No	50%	None	SMOTE	0.63	0.13	0.24	0.04
ECE	No	50%	PCA	SMOTE	0.60	0.14	0.25	0.04
ECE	No	50%	RFE-RF	SMOTE	0.62	0.13	0.25	0.05
ECE	No	50%	Univariate	SMOTE	0.62	0.14	0.25	0.05
ECE	No	55%	None	None	0.70	0.13	0.22	0.04
ECE	No	55%	PCA	None	0.69	0.14	0.23	0.05
ECE	No	55%	RFE-RF	None	0.67	0.13	0.24	0.05
ECE	No	55%	Univariate	None	0.66	0.13	0.24	0.05
ECE	No	55%	None	SMOTE	0.70	0.13	0.22	0.04
ECE	No	55%	PCA	SMOTE	0.70	0.14	0.23	0.05
ECE	No	55%	RFE-RF	SMOTE	0.67	0.14	0.24	0.05
ECE	No	55%	Univariate	SMOTE	0.66	0.14	0.24	0.05
ECE	No	60%	None	None	0.73	0.13	0.21	0.04
ECE	No	60%	PCA	None	0.62	0.13	0.25	0.05
ECE	No	60%	RFE-RF	None	0.76	0.12	0.21	0.05
ECE	No	60%	Univariate	None	0.73	0.13	0.21	0.05
ECE	No	60%	None	SMOTE	0.72	0.13	0.21	0.04
ECE	No	60%	PCA	SMOTE	0.62	0.14	0.25	0.05
ECE	No	60%	RFE-RF	SMOTE	0.76	0.12	0.21	0.05
ECE	No	60%	Univariate	SMOTE	0.72	0.13	0.22	0.05
ECE	No	65%	None	None	0.66	0.13	0.22	0.04
ECE	No	65%	PCA	None	0.65	0.14	0.24	0.04
ECE	No	65%	RFE-RF	None	0.66	0.13	0.23	0.05
ECE	No	65%	Univariate	None	0.68	0.13	0.22	0.05
ECE	No	65%	None	SMOTE	0.66	0.13	0.22	0.04
ECE	No	65%	PCA	SMOTE	0.65	0.14	0.24	0.04
ECE	No	65%	RFE-RF	SMOTE	0.66	0.13	0.23	0.05
ECE	No	65%	Univariate	SMOTE	0.68	0.13	0.22	0.05
ECE	No	70%	None	None	0.76	0.12	0.20	0.04
ECE	No	70%	PCA	None	0.72	0.13	0.21	0.04
ECE	No	70%	RFE-RF	None	0.76	0.12	0.20	0.05
ECE	No	70%	Univariate	None	0.75	0.12	0.21	0.05
ECE	No	70%	None	SMOTE	0.76	0.12	0.20	0.04
ECE	No	70%	PCA	SMOTE	0.72	0.13	0.21	0.04
ECE	No	70%	RFE-RF	SMOTE	0.76	0.12	0.20	0.05
ECE	No	70%	Univariate	SMOTE	0.75	0.12	0.21	0.05
ECE	Yes	50%	None	None	0.64	0.13	0.23	0.04
ECE	Yes	50%	PCA	None	0.60	0.14	0.25	0.04
ECE	Yes	50%	RFE-RF	None	0.64	0.13	0.24	0.05

ECE	Yes	50%	Univariate	None	0.64	0.14	0.23	0.05
ECE	Yes	50%	None	SMOTE	0.63	0.14	0.23	0.04
ECE	Yes	50%	PCA	SMOTE	0.60	0.14	0.25	0.04
ECE	Yes	50%	RFE-RF	SMOTE	0.64	0.13	0.24	0.05
ECE	Yes	50%	Univariate	SMOTE	0.64	0.14	0.23	0.05
ECE	Yes	55%	None	None	0.66	0.13	0.23	0.04
ECE	Yes	55%	PCA	None	0.56	0.14	0.26	0.04
ECE	Yes	55%	RFE-RF	None	0.69	0.13	0.22	0.05
ECE	Yes	55%	Univariate	None	0.67	0.13	0.23	0.05
ECE	Yes	55%	None	SMOTE	0.66	0.13	0.23	0.04
ECE	Yes	55%	PCA	SMOTE	0.57	0.14	0.26	0.04
ECE	Yes	55%	RFE-RF	SMOTE	0.68	0.13	0.22	0.05
ECE	Yes	55%	Univariate	SMOTE	0.67	0.13	0.23	0.05
ECE	Yes	60%	None	None	0.63	0.13	0.24	0.04
ECE	Yes	60%	PCA	None	0.59	0.12	0.26	0.04
ECE	Yes	60%	RFE-RF	None	0.65	0.13	0.23	0.05
ECE	Yes	60%	Univariate	None	0.64	0.14	0.24	0.05
ECE	Yes	60%	None	SMOTE	0.62	0.13	0.24	0.04
ECE	Yes	60%	PCA	SMOTE	0.59	0.12	0.26	0.04
ECE	Yes	60%	RFE-RF	SMOTE	0.65	0.13	0.23	0.05
ECE	Yes	60%	Univariate	SMOTE	0.64	0.14	0.24	0.05
ECE	Yes	65%	None	None	0.69	0.12	0.22	0.04
ECE	Yes	65%	PCA	None	0.64	0.12	0.24	0.04
ECE	Yes	65%	RFE-RF	None	0.68	0.12	0.22	0.04
ECE	Yes	65%	Univariate	None	0.67	0.13	0.23	0.05
ECE	Yes	65%	None	SMOTE	0.69	0.12	0.22	0.04
ECE	Yes	65%	PCA	SMOTE	0.64	0.13	0.24	0.04
ECE	Yes	65%	RFE-RF	SMOTE	0.68	0.12	0.22	0.04
ECE	Yes	65%	Univariate	SMOTE	0.67	0.12	0.23	0.05
ECE	Yes	70%	None	None	0.63	0.13	0.24	0.04
ECE	Yes	70%	PCA	None	0.64	0.13	0.24	0.04
ECE	Yes	70%	RFE-RF	None	0.64	0.13	0.24	0.05
ECE	Yes	70%	Univariate	None	0.63	0.13	0.24	0.05
ECE	Yes	70%	None	SMOTE	0.63	0.13	0.24	0.04
ECE	Yes	70%	PCA	SMOTE	0.65	0.13	0.24	0.04
ECE	Yes	70%	RFE-RF	SMOTE	0.64	0.13	0.24	0.04
ECE	Yes	70%	Univariate	SMOTE	0.63	0.13	0.24	0.05
