# Supplementary material for: Machine learning-based analysis of [18F]DCFPyL PET radiomics for risk stratification in primary prostate cancer
Source: Eur J Nucl Med Mol Imaging. 2020 Jul 31;48(2):340–9. doi: 10.1007/s00259-020-04971-z (PMC7835295; doi:10.1007/s00259-020-04971-z)
Supplement: Supplementary file 4 — (PDF 136 kb). [file 259_2020_4971_MOESM4_ESM.pdf]

Supplementary Table 3A: Feature importance coefficients for LNI prediction. Derived from a Random Forest using univariate feature selection and minority class oversampling, with a 70% delineation threshold and PVC.

Feature type	Feature name	Importance coefficient
intensity volume	difference vol at int fraction	0.136600443
intensity volume	volume at int fraction 10	0.112266973
glcmFeatures2Davg	inverse difference normalised	0.054315009
Intensity histogram	Coefficient of variation	0.053671251
glcmFeatures2Davg	inverse difference moment normalised	0.046424265
gldzmFeatures2Davg	Zone distance variance GLDZM	0.045800423
gldzmFeatures3D	Grey level non uniformity GLDZM	0.033761014
gldzmFeatures2Davg	Large distance emphasis GLDZM	0.030633368
gldzmFeatures2Dmrg	Zone distance variance GLDZM	0.027328423
GLSZMFeatures3D	Grey level non uniformity GLSZM	0.027304853
Morphology	approximate volume	0.024483378
glcmFeatures2Dmrg	cluster shade	0.023883918
glcmFeatures2Davg	cluster shade	0.023305511
GLRLMFeatures3Dmrg	Run length non uniformity	0.02007559
Intensity histogram	10th percentile	0.019741301
Morphology	Surface	0.019490253
Morphology	Volume	0.019428521
glcmFeatures2Davg	joint entropy	0.018250071
GLRLMFeatures3Davg	Run length non uniformity	0.01616803
intensity volume	int at vol fraction 90	0.015686775
glcmFeatures2Davg	sum entropy	0.013439035
gldzmFeatures2Davg	Large distance high grey level emphasis GLDZM	0.012693266
gldzmFeatures2Davg	Zone distance non uniformity GLDZM	0.011485314
GLRLMFeatures2DDmrg	Run length non uniformity	0.010814781
GLRLMFeatures2Dvmrg	Run length non uniformity	0.010659865
GLRLMFeatures2Davg	Run length non uniformity	0.010095309
Statistics	Energy	0.010011034
gldzmFeatures2Dmrg	Large distance emphasis GLDZM	0.009799135
gldzmFeatures2Dmrg	Zone distance non uniformity GLDZM	0.009668087
GLSZMFeatures2Davg	Zone size non uniformity	0.009401004
ngldmFeatures2Davg	Dependence count entropy	0.009197788
Morphology	least axis length	0.008673824
Intensity histogram	Energy	0.008506085
GLRLMFeatures2DWmrg	Run length non uniformity	0.008351132
Statistics	10th percentile	0.007794818
ngldmFeatures3Dmrg	Dependence count non uniformity	0.007288046
GLRLMFeatures2DWmrg	Run entropy	0.007287639
ngldmFeatures3Dmrg	High dependence high grey level emphasis	0.007285564
ngldmFeatures2Davg	Dependence count non uniformity	0.00714149
Morphology	integrated intensity	0.006469414
GLSZMFeatures2Dvmrg	Zone size non uniformity	0.00638641
GLSZMFeatures2Davg	Zone size entropy	0.006234788
GLSZMFeatures3D	Zone size non uniformity	0.006113085
GLRLMFeatures2Davg	Run entropy	0.006089305
gldzmFeatures3D	Zone distance non uniformity GLDZM	0.005567668
ngldmFeatures2Dmrg	Dependence count non uniformity	0.005396625
gldzmFeatures2Davg	Zone distance entropy GLDZM	0.004991901
Local intensity	local intensity peak	0.004538219

Supplementary Table 3B: Feature importance coefficients for metastasis prediction. Derived from a Random Forest using univariate feature selection and minority class oversampling, with a 70% delineation threshold and PVC.

Feature type	Feature name	Importance coefficient
intensity volume	volume at int fraction 10	0.114668
intensity volume	difference vol at int fraction	0.106269
gldzmFeatures3D	Grey level non uniformity GLDZM	0.040154
GLRLMFeatures3Davg	Run length non uniformity	0.034138
GLRLMFeatures3Dmrg	Run length non uniformity	0.032877
Intensity histogram	Coefficient of variation	0.032826
Morphology	Volume	0.031644
Morphology	approximate volume	0.030623
GLSZMFeatures3D	Grey level non uniformity GLSZM	0.030374
gldzmFeatures2Davg	Large distance emphasis GLDZM	0.029148
GLRLMFeatures2DDmrg	Run length non uniformity	0.028139
GLRLMFeatures2Dvmrg	Run length non uniformity	0.027808
glcmFeatures2Davg	joint entropy	0.027348
gldzmFeatures2Davg	Zone distance variance GLDZM	0.027256
Morphology	Surface	0.023731
Morphology	integrated intensity	0.019636
gldzmFeatures2Dmrg	Zone distance non uniformity GLDZM	0.019633
GLSZMFeatures2Dvmrg	Zone size non uniformity	0.018246
intensity volume	int at vol fraction 90	0.017749
gldzmFeatures2Dmrg	Zone distance variance GLDZM	0.015726
Intensity histogram	10th percentile	0.015436
glcmFeatures2Davg	sum entropy	0.015305
GLSZMFeatures2Davg	Zone size non uniformity	0.014166
gldzmFeatures2Davg	Zone distance non uniformity normalized GLDZM	0.013716
ngldmFeatures2Dmrg	Dependence count non uniformity	0.012965
gldzmFeatures2Davg	Zone distance non uniformity GLDZM	0.01261
ngldmFeatures3Dmrg	Dependence count non uniformity	0.011983
Statistics	Energy	0.011837
Statistics	10th percentile	0.01165
Morphology	least axis length	0.0111
ngldmFeatures3Dmrg	Dependence count entropy	0.011002
gldzmFeatures2Dmrg	Large distance emphasis GLDZM	0.010838
ngldmFeatures2Davg	Dependence count non uniformity	0.010634
GLRLMFeatures2DWmrg	Run length non uniformity	0.010036
gldzmFeatures2Davg	Zone distance entropy GLDZM	0.009802
ngldmFeatures2Davg	Dependence count entropy	0.009773
GLSZMFeatures3D	Zone size non uniformity	0.009769
glcmFeatures2DDmrg	joint entropy	0.009369
gldzmFeatures2Davg	small distance emphasis GLDZM	0.009272
GLRLMFeatures2Davg	Run length non uniformity	0.009235
Local intensity	local intensity peak	0.009139
GLRLMFeatures2DWmrg	Run entropy	0.0088
Intensity histogram	Energy	0.008626
glcmFeatures2Dmrg	joint entropy	0.008183
gldzmFeatures3D	Zone distance non uniformity GLDZM	0.007646
glcmFeatures3Davg	joint entropy	0.006533
GLRLMFeatures2Davg	Run entropy	0.006489
GLSZMFeatures2Davg	Zone size entropy	0.006089

Supplementary Table 3C: Feature importance coefficients for Gleason score ≥8 prediction. Derived from a Random Forest using univariate feature selection and minority class oversampling, with a 60% delineation threshold and PVC.

Feature type	Feature name	Importance coefficient
GLSZMFeatures2Dvmrg	Zone size non uniformity	0.069759
gldzmFeatures3D	Zone distance non uniformity GLDZM	0.057953
gldzmFeatures2Davg	Grey level variance GLDZM	0.047418
glcmFeatures3Davg	joint entropy	0.039737
ngldmFeatures2Dmrg	Dependence count non uniformity	0.035236
glcmFeatures2Dmrg	joint entropy	0.03356
gldzmFeatures2Davg	Zone distance entropy GLDZM	0.033075
glcmFeatures2Dmrg	sum entropy	0.032995
glcmFeatures2DDmrg	joint entropy	0.029689
gldzmFeatures2Dmrg	Zone distance non uniformity GLDZM	0.027118
GLRLMFeatures2DWmrg	Run entropy	0.027041
gldzmFeatures2Davg	small distance emphasis GLDZM	0.025977
GLSZMFeatures2Davg	Zone size entropy	0.025013
gldzmFeatures2Davg	Zone distance non uniformity normalized GLDZM	0.024586
ngldmFeatures2Davg	Dependence count entropy	0.023535
GLRLMFeatures2Davg	Run entropy	0.022961
Intensity histogram	Minimum histogram gradient grey level	0.022603
glcmFeatures2Davg	sum entropy	0.02195
GLRLMFeatures2DDmrg	Run length non uniformity	0.021553
glcmFeatures2Davg	difference entropy	0.020787
ngldmFeatures3Dmrg	Dependence count non uniformity	0.019981
GLRLMFeatures2Dvmrg	Run length non uniformity	0.018537
ngtdmFeatures2Dmrg	coarseness	0.017589
ngldmFeatures2Davg	Dependence count non uniformity	0.017289
GLSZMFeatures3D	Zone size non uniformity	0.01715
gldzmFeatures2Davg	Zone distance non uniformity GLDZM	0.01714
GLRLMFeatures2Davg	Run length non uniformity	0.016217
glcmFeatures2Davg	joint entropy	0.016118
gldzmFeatures3D	Grey level non uniformity GLDZM	0.014434
GLSZMFeatures3D	Zone size entropy	0.014335
GLRLMFeatures2DWmrg	Run length non uniformity	0.013717
gldzmFeatures2Davg	Large distance emphasis GLDZM	0.013479
GLRLMFeatures3Dmrg	Run length non uniformity	0.013443
Morphology	integrated intensity	0.013369
GLSZMFeatures3D	Grey level non uniformity GLSZM	0.012678
Morphology	Surface to volume ratio	0.011909
GLSZMFeatures2Davg	Zone size non uniformity	0.011537
ngldmFeatures3Dmrg	Dependence count entropy	0.011408
GLRLMFeatures3Davg	Run length non uniformity	0.011324
glcmFeatures2Dvmrg	joint entropy	0.009972
intensity volume	int at vol fraction 90	0.009951
Intensity histogram	10th percentile	0.009473
glcmFeatures3Davg	sum entropy	0.009391
ngtdmFeatures3D	coarseness	0.009085
ngldmFeatures2Dmrg	Dependence count entropy	0.007755
ngldmFeatures3Dmrg	dependence Count Energy	0.007533
gldzmFeatures2Dmrg	small distance emphasis GLDZM	0.007212
gldzmFeatures2Dmrg	Zone distance entropy GLDZM	0.005428

Supplementary Table 4C: Feature importance coefficients for ECE prediction. Derived from a Random Forest using recursive feature elimination (top 10% features) and minority class oversampling, with a 60% delineation threshold and no PVC.

Feature type	Feature name	Importance coefficient
intensity volume	difference vol at int fraction	0.028414476
GLSZMFeatures3D	Grey level non uniformity GLSZM	0.023252986
intensity volume	volume at int fraction 10	0.022882143
gldzmFeatures3D	Grey level non uniformity GLDZM	0.017863573
GLRLMFeatures2Dvmrg	Run length non uniformity	0.016115545
GLRLMFeatures2DDmrg	Run length non uniformity	0.015842647
gldzmFeatures2Dmrg	Zone distance non uniformity GLDZM	0.014690522
ngldmFeatures2Dmrg	Dependence count non uniformity	0.014386668
GLRLMFeatures3Davg	Run length non uniformity	0.012159532
ngldmFeatures3Dmrg	Dependence count non uniformity	0.010899154
GLRLMFeatures3Dmrg	Run length non uniformity	0.010864486
glcmFeatures2Davg	inverse difference moment normalised	0.010781868
glcmFeatures2Davg	inverse difference normalised	0.010514871
Morphology	Gearys C	0.010321328
GLRLMFeatures2Davg	Run length non uniformity	0.010097738
Morphology	minor axis length	0.009974725
glcmFeatures3Davg	inverse difference normalised	0.009441387
glcmFeatures3DWmrg	inverse difference normalised	0.009202084
glcmFeatures3DWmrg	inverse difference moment normalised	0.008932004
gldzmFeatures2Dmrg	Zone distance non uniformity normalized GLDZM	0.007899039
ngtdmFeatures3D	coarseness	0.007662809
GLRLMFeatures2DWmrg	Run length non uniformity	0.00747051
glcmFeatures3Davg	inverse difference moment normalised	0.007406827
gldzmFeatures3D	Zone distance non uniformity GLDZM	0.007244934
GLSZMFeatures2Dvmrg	Zone size non uniformity	0.006957598
glcmFeatures2Dvmrg	inverse difference moment normalised	0.006920687
gldzmFeatures2Dmrg	small distance emphasis GLDZM	0.006687897
Morphology	maximum 3D diameter	0.006534258
glcmFeatures2Dvmrg	inverse difference normalised	0.006179427
ngtdmFeatures2avg	coarseness	0.006097922
glcmFeatures3Davg	correlation	0.005941971
gldzmFeatures3D	Zone distance variance GLDZM	0.005931324
intensity volume	volume at int fraction 90	0.005835955
gldzmFeatures2Davg	small distance emphasis GLDZM	0.005835417
glcmFeatures2DDmrg	inverse difference normalised	0.005773739
Morphology	Surface to volume ratio	0.005680252
ngldmFeatures2Davg	Dependence count variance	0.005578475
glcmFeatures2DDmrg	inverse variance	0.005561389
Morphology	approximate volume	0.005533079
Morphology	Surface	0.005475786
glcmFeatures2Davg	angular second moment	0.005439508
gldzmFeatures2Davg	Zone distance variance GLDZM	0.005392424
ngldmFeatures2Davg	dependence Count Energy	0.005317093
ngtdmFeatures2Dmrg	coarseness	0.005233675
gldzmFeatures2Dmrg	Grey level non uniformity GLDZM	0.005233215
glcmFeatures2Dvmrg	first measure of information correlation	0.00513463
glcmFeatures3DWmrg	correlation	0.005101676
Morphology	flatness	0.004970668
